# Neurophysiological, Radiological, and Molecular Biomarkers of Pain-Related Conditions: An Umbrella Review

**DOI:** 10.3390/jcm15020550

**Published:** 2026-01-09

**Authors:** Dmitriy Viderman, Sultan Kalikanov, Diyara Mukazhan, Bermet Nurmukhamed

**Affiliations:** 1 Department of Surgery, School of Medicine, Nazarbayev University, Astana 010000, Kazakhstan; sultan.kalikanov@alumni.nu.edu.kz (S.K.);; 2 Department of Anesthesiology, Intensive Care, and Pain Medicine, National Research Oncology Center, Astana 010000, Kazakhstan

**Keywords:** chronic pain, biomarker, inflammatory and neurophysiological markers

## Abstract

**Background/Objectives**: Pain and pain-related conditions are considered a global health and financial burden. In order to improve pain management, pain intensity assessment, and pain diagnosis, various biomarkers have been proposed. Since their clinical utility is not proven yet, the aim of this umbrella review is to synthesize existing evidence of all types of pain biomarkers available. **Methods**: Systematic searches were conducted in PubMed, Scopus, and the Cochrane Library from inception to 2 June 2025. Eligible studies were systematic reviews and meta-analyses examining any clinical, biochemical, genetic, neurophysiological, or imaging biomarker related to pain. The screening of studies, data extraction, and assessment of methodological quality using the AMSTAR-2 tool were conducted by two independent reviewers. Findings were summarized narratively. **Results**: A total of 49 systematic reviews and meta-analyses were included. Most reviews were rated as low or critically low quality. Inflammatory biomarkers (CRP, IL-6, TNF-α) reported the most consistent associations with chronic musculoskeletal pain, while neuroimaging and EEG measures reflected central nervous system alterations. Proteomic multi-protein panels demonstrated exploratory diagnostic potential, particularly for fibromyalgia, but lacked clinical validation. Evidence for genetic, hormonal, metabolic, neurochemical, and tissue-specific biomarkers was inconsistent and methodologically limited, supporting mechanistic rather than clinical inference. **Conclusions**: No single biomarker has achieved clinical validation for chronic pain, but several biomarker classes show promise. Future implications include high-quality longitudinal studies, standardized protocols, and multidimensional biomarker panels.

## 1. Introduction

Chronic pain as a non-communicable disease is a global concern; it poses a major burden on the health of the people and the health care system. According to a meta-analysis that collected data on the worldwide burden of diseases in Europe, Asia, and the United States and included 25 studies, the prevalence of chronic pain is around 20% [1]. Chronic pain has multiple origins that are biological, psychological, and socio-demographic. It is caused by injury or disease and may involve musculoskeletal conditions such as arthritis, back pain, or fibromyalgia, and neuropathic conditions such as diabetic neuropathy or post-herpetic neuralgia [2,3]. The prevalence of LBP increases with age, and women have been found to have more severe as well as widespread pain than men [4,5]. Several patient or disease factors cause the burden to be higher, including lower income and education, and a history of abuse or trauma. Smoking and lack of physical activity also play a part in chronic pain as a lifestyle factor [6].

The burden of chronic pain extends beyond physical suffering. Pain’s economic consequence is more extensive than that of most other illnesses, mainly because of its cost-effect on productivity, which involves high absence from work rates, low activity levels, and early retirement [7,8]. For example, anxiety caused by pain, especially during flare-ups, was depicted in the US study, where arthritis would cost $7.1 billion in lost productive work time, with 65.7% of this cost attributed to the 38% of workers experiencing pain exacerbations [9].

There is a pressing need for better tools to assess, monitor, and manage chronic pain. Biomarkers have been suggested as one such tool; however, several challenges remain in their clinical application. The heterogeneity of pain conditions complicates the identification of universally applicable biomarkers. Furthermore, the transition from laboratory research to clinical practice is often hindered by issues related to specificity, accessibility, and the need for standardized protocols [10]. As a result, while some biomarkers have shown promise, many are still in the exploratory phase, requiring further validation through large-scale studies that account for confounding factors and variability in pain presentations [11,12].

New directions in pain management focus on the application of the principles of pharmacogenomics, epigenomics, and proteomics as a roadmap to creating individualized treatment strategies for patients with chronic pain. Neuroimaging has also enhanced knowledge of the perception and sustained nature of chronic pain and its neural basis. Pharmacological, psychological, and physical methods are also increasingly regarded as important intervention techniques. Nevertheless, there are some issues concerning the application of these findings into routine clinical practice, which supports the necessity of further interdisciplinary cooperation and a patient-centered approach to this complex and widespread issue.

A clinically useful biomarker would require a consistent association with pain-related outcomes, analytical reliability, and external validation. Recent years have seen rapid growth in biomarker research for chronic pain, accompanied by an increasing number of systematic reviews across diverse biomarker classes and pain conditions. Nevertheless, this evidence remains fragmented, with substantial variation in scope, methodology, outcome definitions, and conclusions. This limits comparability and obscures consistent patterns. Many reviews focus on single biomarker domains or specific conditions, further restricting broader interpretation. Additionally, the methodological quality of published reviews varies widely, and conclusions often do not adequately account for review-level limitations. Therefore, the clinical relevance of largely exploratory biomarkers may be overstated. This umbrella review synthesizes evidence from existing systematic reviews on pain biomarkers. It aims to summarize the current findings, distinguish mechanistic insight from clinical applicability, and identify areas in the literature that require further research.

## 2. Materials and Methods

### 2.1. Search Strategy

We followed the PRISMA (Preferred Reporting Items for Systematic Reviews and Meta-Analyses) [13] guidelines during the execution of this review, supplemented by recommendations for conducting systematic reviews of systematic reviews. The PRISMA checklist is provided in the Supplementary Materials (Table S1: PRISMA checklist). The protocol was registered in the Open Science Framework (DOI 10.17605/OSF.IO/NDRPV). A predesigned search strategy was used to conduct a systematic search in the PubMed, Scopus, and Cochrane Library databases from the inception until 2 June 2025.

The search terms used were:

Cochrane library:

(“systematic review”:ti,ab,kw OR “meta-analysis”:ti,ab,kw)

AND (“pain”:ti,ab,kw OR “chronic pain”:ti,ab,kw)

AND (“biomarker”:ti,ab,kw OR “biological marker”:ti,ab,kw)

PubMed

((“systematic review”[Publication Type] OR “meta-analysis”[Publication Type]

OR “systematic review”[Title/Abstract] OR “meta-analysis”[Title/Abstract])

AND (“pain”[Title/Abstract] OR “chronic pain”[Title/Abstract])

AND (“biomarker”[Title/Abstract] OR “biological marker”[Title/Abstract] OR “Biological Markers”[MeSH]))

Scopus

(TITLE-ABS-KEY(“systematic review” OR “meta-analysis”)

AND TITLE-ABS-KEY(pain OR “chronic pain”)

AND TITLE-ABS-KEY(“biomarker” OR “biological marker”))

### 2.2. Inclusion and Exclusion Criteria

Inclusion Criteria were:

Study type: Systematic reviews (with or without meta-analysis).

Population/Condition: Patients with pain, including chronic pain, across any disease or condition.

Intervention/Exposure: Biomarkers were defined as objectively measured biological, imaging, or neurophysiological indicators associated with pain. Biomarkers may be clinical, biochemical, or radiological/imaging. Clinical validation was not required for inclusion, and biomarkers were considered irrespective of their stage of translational development.

Comparators: Any comparator or none (different biomarkers, usual clinical assessment, placebo, or no comparator).

Outcomes: Reported role of biomarkers in the detection, assessment, or monitoring of pain.

Language: English. The translation of non-English reviews was not feasible within the resources of the project. This may introduce language bias.

Exclusion Criteria were:

Primary studies (RCTs, observational studies).

Articles where biomarkers are not studied in relation to pain.

### 2.3. Literature Screening and Data Extraction

Two reviewers underwent calibration training to ensure consistent interpretation of the eligibility criteria and biomarker definitions. Data extraction was conducted independently by two reviewers using a predefined and pilot-tested data extraction form. They first reviewed titles and abstracts, then assessed the full texts. The results of the literature screening were compared, and disagreements at any stage of screening or data extraction were resolved through discussion and consensus between the two reviewers. If consensus could not be reached, a third reviewer was consulted to adjudicate the decision. The process was conducted using standard reference management and spreadsheet software (Word and Excel). The data extraction process encompassed various parameters including author, country, year, citations, study design (SR&MA of RCTs or observational studies), goals of the SR, diseases and conditions (in which biomarkers were studied), brief characteristics of patients, biomarkers (clinical, biochemical, radiological), number of patients included in the SR, total number of studies included in the SR, effect of biomarkers on decision-making and outcomes (were they useful in diagnosis, prescription of treatment and improvement of outcomes?), benefits of studied biomarkers, limitation of studied biomarkers, future directions and opportunities, and study conclusions and comments.

Given the heterogeneity of population, interventions, and biomarkers assessed, a narrative synthesis approach was used to summarize and compare findings across reviews.

### 2.4. Methodological Quality Evaluation of Included Studies

Two authors independently assessed the included studies using the 16 items of the AMSTAR-2 tool [14]. Overall confidence ratings were assigned according to the AMSTAR-2 rule of thumb for critical domains: items 2, 4, 7, 9, 11, 13, and 15. Reviews with no or only one non-critical weakness and no critical flaws were rated as high confidence; those with more than one non-critical weakness but no critical flaws were rated as moderate confidence. Reviews with one critical flaw, even if all other domains were adequate, were rated as low confidence, while those with two or more critical flaws were rated as critically low confidence. If a meta-analysis was not conducted, items 11–15 were coded as “not applicable” and not penalized. In cases where supplementary data could not be accessed, the corresponding item was considered unmet.

## 3. Results

**Figure 1:**
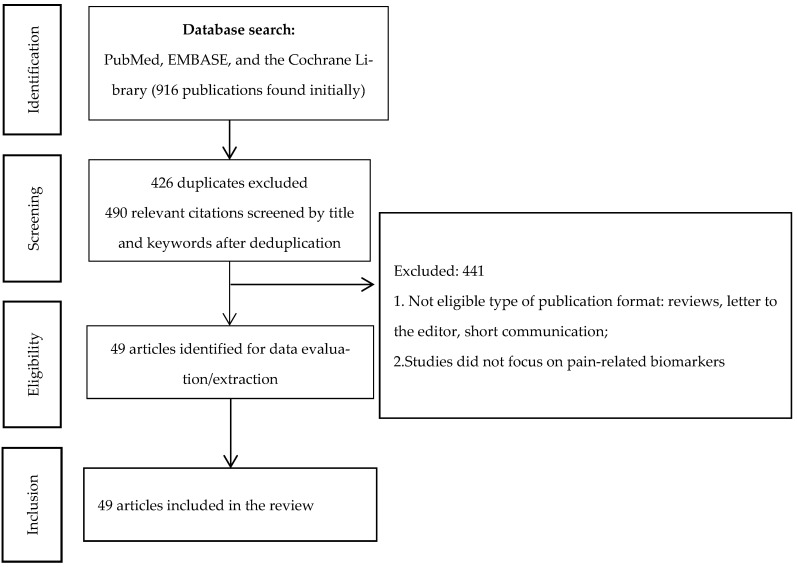
PRISMA flow diagram.

We originally found 916 publications. Of them, 426 duplicates were removed, 490 publications were screened, and 441 publications did not match the criteria. We finally included 49 systematic reviews and meta-analyses [15,16,17,18,19,20,21,22,23,24,25,26,27,28,29,30,31,32,33,34,35,36,37,38,39,40,41,42,43,44,45,46,47,48,49,50,51,52,53,54,55,56,57,58,59,60,61,62,63] that together covered the results of 1369 studies. Of these, 1279 studies were human-only studies, whereas 105 studies involved both human and animal models. The total number of patients reported in these studies was 269,768 (10 SRs did not report the number of patients or involved animal models). The process of database search and consequent exclusion and inclusion of studies is depicted in Figure 1.

Below, we present the key findings categorized by patient characteristics, diseases/conditions, and the various types of biomarkers studied.

### 3.1. Patients’ Characteristics

Table 1 provides an overview of the following characteristics—study design, goals, diseases/conditions, brief characteristics of patients, biomarkers’ name, effect, benefits, and limitations, number of patients, number of the original studies included, future directions, and conclusions—of the included systematic reviews.

### 3.2. Diseases/Conditions

We examined systematic reviews and meta-analyses (SRs and MAs) that reported on the use of biomarkers across a wide range of pain-related diseases and conditions provided in Table 1. These included low back pain (LBP) (16 studies), fibromyalgia (10 studies), osteoarthritis (OA) (8 studies), migraine (5 studies), peripheral neuropathies (4 studies), endometriosis (2 studies), ankylosing spondylitis (AS) (3 studies), cancer-induced pain (3 times), multiple sclerosis (MS) (2 time), trigeminal pain (2 studies), rheumatoid arthritis (RA) (2 studies), bladder pain syndrome (2 studies), cluster headache (2 studies), shoulder disorders (2 studies), irritable bowel syndrome (2 studies), temporomandibular disorders (2 studies), and pancreatitis (2 studies). The following conditions were reported only once: spinal cord injury, somatoform pain disorder, type 1 diabetes–related neuropathy, phantom limb pain, musculoskeletal pain, interstitial cystitis, myofascial pain syndrome, delayed onset muscle soreness, sciatica, whiplash, lateral epicondylalgia, lumbar radicular pain, adhesive capsulitis, trapezius myalgia, central fatigue syndrome, chikungunya virus infection, pediatric musculoskeletal pain, neonatal procedural pain, and central post-stroke pain.

### 3.3. Geographical Coverage

The systematic reviews and meta-analyses included in this study reported the prevalence of pain among patients from a wide array of geographical regions and countries, as detailed in Table 1. Specific countries included Portugal (2 studies), the USA (6 studies), Germany (4 studies), Spain (5 studies), France (1 study), Switzerland (2 studies), Brazil (3 studies), the Netherlands (3 studies), the UK (2 studies), Australia (4 studies), China (4 studies), Canada (2 studies), Italy (1 study), Norway (1 study), Sweden (1 study), Scotland (1 study), Poland (1 study), Colombia (1 study), Greece (1 study), Denmark (1 study), and Hong Kong (1 study).

### 3.4. Methodological Quality Assessment

Based on the AMSTAR-2 analysis in Table 2, 1 study was found to be high-quality [62], 1 study was moderate-quality [58], 5 studies were low-quality [16,20,39,47,59], and 42 studies were critically low-quality [15,17,18,19,21,22,24,25,26,27,28,29,30,31,32,33,34,35,36,37,38,40,41,43,44,45,46,48,49,50,51,52,53,54,55,56,57,60,61,63]. One study [23] was not assessed for methodological quality as AMSTAR-2 is not applicable to narrative reviews. The AMSTAR assessment of the included studies reveals variability in methodological quality. Common strengths include clearly articulated research questions and literature search strategies. However, many studies lacked justification for the selection of study designs and did not report excluded studies. Techniques for assessing the risk of bias were satisfactory in some studies but inconsistent overall. Meta-analyses generally adhered to appropriate statistical methods, but assessments of the potential impact of bias were less frequently reported. Improvements are needed in the investigation and explanation of heterogeneity and publication bias. While many studies declared conflicts of interest, this was not universally observed.

### 3.5. Biomarkers of Pain

The results are organized according to biomarker type. The synthesis of findings is presented hierarchically, prioritizing evidence derived from human studies over non-human studies. In this umbrella review, the interpretation of biomarker evidence is based on the conclusions reported by the included systematic reviews, as summarized in Table 1, reflecting how those reviews interpreted their findings according to their respective analyses. No independent grading of biomarker evidence strength or clinical validity was performed. The aim was to highlight the key biomarkers identified and discuss their potential clinical implications.

### 3.6. Immune System Biomarkers

#### 3.6.1. Evidence Reported as Consistent in the Included Systematic Reviews

There is consistent evidence of an association between chronic pain conditions and inflammation. It was found that specific pro-inflammatory markers are consistently elevated in chronic pain. For example, studies on non-specific low back pain (NsLBP) and chronic low back pain revealed that elevated levels of C-reactive protein (CRP), Interleukin-6 (IL-6), and Tumor Necrosis Factor-alpha (TNF-α) may be associated with the presence and pain severity [25,51,52,56]. Furthermore, CRP, IL-6, and TNF-α were found to be reduced by resistance and neuromuscular training in osteoarthritis (OA) patients with pain caused by physical dysfunction [47]. In addition to NsLBP and OA, inflammatory markers may have shown prognostic value in the context of Chikungunya virus infection. IL-6, IL-4, and CRP may work as risk indicators of developing joint pain and chronic arthritis after the infection’s acute phase [48]. Finally, one review found that the difference in levels of IL-6, IL-8, TNF-α, and brain-derived neurotrophic factor (BDNF) may have the potential to be used for stratification of patients with fibromyalgia into subgroups for future targeted therapies [21].

#### 3.6.2. Evidence Reported as Limited or Inconclusive in the Included Systematic Reviews

Even though the association between inflammatory biomarkers and pain is promising, there is limited clinical utility. There are several reviews with very low to low-level evidence of a clear association between pain intensity and inflammatory biomarkers [16,27,36,37,43,57]. These reviews also lack longitudinal data, which makes it difficult to identify whether the inflammation is a cause or a consequence of chronic pain. One meta-analysis on diabetic peripheral neuropathy (DPN) showed that TNF-α can differentiate painful from non-painful DPN. However, the results were limited by heterogeneity in study design [22]. In other reviews, inflammatory biomarkers provided insights into the pathological processes of chronic regional pain syndrome (CRPS) Type I [20], idiopathic frozen shoulder [31], OA [45], and sciatica [33]. Nevertheless, these reviews reported a small sample size, confounding by other diseases, and a lack of standardized diagnostic criteria.

### 3.7. Proteomic Biomarkers

#### 3.7.1. Evidence Reported as Consistent in the Included Systematic Reviews

Besides inflammatory biomarkers, biomarker research is shifting towards the analysis of multi-protein panels. One systematic review demonstrated promising diagnostic accuracy in distinguishing healthy people from patients with fibromyalgia. Such panels of proteins as transferrin, α2-macroglobulin, and specific immunoglobulin fractions, when used in conjunction with decision tree models, may be more accurate than a single marker [50].

#### 3.7.2. Evidence Reported as Limited or Inconclusive in the Included Systematic Reviews

Other studies that investigated proteomic biomarkers yielded unvalidated findings. One scoping review found an increased level of fecal and urinary markers (macrophage migration inhibitory factor (MIF) and fecal glyceraldehyde) in patients with bladder pain syndrome (BPS). It was noted that this correlation lacked validation [29]. Another review compared myofascial pain syndrome (MPS) and delayed onset muscle soreness (DOMS) and identified several useful biomarkers for diagnosis. Nevertheless, most of the studies were heterogeneous [26]. A systematic review on cancer-induced bone pain did not conclude any genetic clinical biomarker [34].

#### 3.7.3. Animal Models

In this umbrella review, one systematic review of animal studies showed how physiotherapy may modulate neurotrophins, neurotransmitters, and cytokines [18]. One systematic review that involved human and animal studies helped understand the pathogenesis and mechanisms of endometriosis pain, but lacked RCTs with a large sample size [32]. Non-human studies may provide a foundation for understanding the mechanism of action in order to inform future human trials.

### 3.8. Structural and Functional Neuroimaging Biomarkers

#### 3.8.1. Evidence Reported as Consistent in the Included Systematic Reviews

Since chronic pain is a condition associated with the central nervous system, there might be evidence in neuroimaging studies. Trigeminal neuralgia was investigated in one review. The study found consistent brain changes in the thalamus, cingulate, and insula [28]. A meta-analysis on chronic low back pain (CLBP) found significant gray matter volume changes. Specifically, the volume was decreased in the left superior frontal gyrus, left insula, and right striatum and increased in the left striatum and left post-central gyrus [54]. These regions are involved in the reward mechanism, emotions, but most importantly, in pain processing. Thus, such neuroimaging signatures may be proposed as potential biomarkers for chronic pain development.

#### 3.8.2. Evidence Reported as Limited or Inconclusive in the Included Systematic Reviews

The clinical utility of neuroimaging is not yet fully realized due to limitations in several studies. One systematic review found only limited evidence that reduced neck muscle size can be used as a quantitative imaging biomarker for neck and shoulder pain [40]. Another review proposed an association between high-intensity zones (HIZs) and low back pain. The study was limited by a small sample size and the absence of standardized protocols [35]. Next, two systematic reviews provided data on lesion locations and underlying mechanisms confirmed by neuroimaging, but the development of biomarkers for prognosis has not been proposed yet [46,63]. Finally, an inconsistent correlation between magnetic resonance imaging (MRI) and OA patients’ reported pain was found in another review [42].

### 3.9. Neurophysiological Testing

#### 3.9.1. Evidence Reported as Consistent in the Included Systematic Reviews

EEG is a non-invasive procedure that may identify electrical patterns with high temporal resolution. Several systematic reviews reported consistent resting-state EEG pattern changes in chronic neuropathic pain. EEG showed patterns of increase in theta power and a decrease in alpha and beta power [15,19]. Moreover, meta-analysis found that gamma-band oscillations (GBOs) may be positively correlated with subjective pain [59]. Specifically, the frequency and spatial location of GBOs varied with the pain type. Phasic (acute) pain-induced GBOs were higher in frequency (~66 Hz) and localized to the sensorimotor cortex. Chronic pain-associated GBOs were lower in frequency (~55 Hz) and predominantly localized over the prefrontal cortex [59].

#### 3.9.2. Evidence Reported as Limited or Inconclusive in the Included Systematic Reviews

Other EEG measures were limited by study methodology to clearly identify reliable biomarkers [17,60]. One review showed that patients with chronic pain may decrease the threshold for activation of defensive reflexes in comparison to healthy patients [38]. This suggests an increased need for body protection. Nonetheless, the review reported high heterogeneity and issues with missing data.

### 3.10. Neurochemical Biomarkers

#### 3.10.1. Evidence Reported as Consistent in the Included Systematic Reviews

Such a neurochemical biomarker as calcitonin gene-related peptide (CGRP) was shown to be consistently elevated in patients with headache attacks in one systematic review [55]. This may make CGRP a promising marker. Likewise, CGRP may be targeted by therapies for migraine and cluster headache management [55,61].

#### 3.10.2. Evidence Reported as Limited or Inconclusive in the Included Systematic Reviews

Other neurochemicals only provided limited and conflicting evidence. A scoping review stated that beta-endorphin as a biomarker for chronic pain has limited evidence for clinical use due to small sample sizes, limited follow-up times, and lack of control groups [23].

#### 3.10.3. Animal Models

In the context of neurochemical biomarkers, one systematic review with preclinical animal models has provided insight into the intervention mechanisms. It reported that physiotherapy could modulate the opioid system (endorphins, receptors) and neurotransmitter substance P [18]. This may provide a biological rationale for how physiotherapy alleviates pain on a cellular level.

### 3.11. Genetic Biomarkers

Evidence Reported as Limited or Inconclusive in the Included Systematic Reviews

One systematic review found that some genetic variants, like OPRM1 and COMT genes, may be linked to poor recovery, but the evidence was limited by heterogeneity, small cohort studies, and the absence of combined analysis of genetic biomarkers and clinical data [39].

### 3.12. Hormonal and Metabolic Biomarkers

#### 3.12.1. Evidence Reported as Consistent in the Included Systematic Reviews

The studies focused on hormonal and metabolic biomarkers reflect the multisystemic nature of chronic pain. One study reviewed burning mouth syndrome (BMS) and found a significant increase in salivary cortisol when compared to controls [53]. Salivary cortisol was also recommended as a promising biomarker for anxiety [53]. Salivary biomarkers were also proposed for monitoring and predicting orthodontic treatment stages [49]. In the same way, changes in the level of metabolites like amino acids, lipids, and carbohydrates have been reported to have an association with chronic pain. However, these metabolites could not be validated for clinical use due to study heterogeneity and a lack of large-scale validation studies [24].

#### 3.12.2. Evidence Reported as Limited or Inconclusive in the Included Systematic Reviews

Evidence on other hormonal and metabolic biomarkers is inconclusive. Particularly, a systematic review on hypothalamic–pituitary–adrenal (HPA) axis biomarkers in fibromyalgia reported high heterogeneity and a lack of consistent patterns, making it unreliable for diagnosis as of now [58]. Another study investigated neonatal pain and found that cortisol level changes with pain and analgesia, but its variability was too great to conclude it a reliable biomarker for clinical practice [62]. Thus, for now, behavioral scales remain the primary tool.

### 3.13. Tissue-Specific Biomarkers

Evidence Reported as Limited or Inconclusive in the Included Systematic Reviews

Several included studies investigated biomarkers for a specific tissue part. This involved joint (OA), skin (CRPS, frozen shoulder), and endogenous pain inhibitory pathways. All of the studies lack full clinical validation.

One review on hip OA identified urinary CTX-II and serum CRP and COMP, but these molecular biomarkers were not adequately validated [44]. Next, a systematic review on MRI biomarkers for patients with OA found only inconsistent results for the quantitative cartilage morphometry technique as a total knee replacement outcome prognostic tool [46]. Another review that investigated knee OA concluded six potential phenotypes but reported a lack of standard phenotype definitions [41].

Regarding CRPS and frozen shoulder, it was found that nerve fiber density could provide insights into the pathophysiology of the diseases. The main limitation was the lack of standardized diagnostic criteria and the confounding caused by prior interventions or surgeries [20].

Conditioned pain modulation (CPM), a measure of endogenous pain inhibitory pathways, was also studied as a potential biomarker for chronic pain diagnosis [30]. The results did not show a promising correlation.

## 4. Discussion

This review aimed to explore various biomarkers associated with pain disorders to understand their application and challenges in clinical settings. To our knowledge, no prior umbrella review has comprehensively addressed this topic.

It was found that the recurrent association of IL-6, TNF-α, and CRP with pain severity across musculoskeletal conditions like OA and LBP [25,47,51,52,56] suggests that they may represent a common inflammatory pain phenotype. It may mean that pain functions as a driver for the inflammatory response and that low-grade inflammation could be a major mechanism responsible for chronic pain development, regardless of the initial cause. Additionally, inflammatory biomarkers may have utility for early diagnosis in various contexts like fibromyalgia and even Chikungunya virus infection [21,48]. Nevertheless, the clinical utility is often limited by small sample sizes, methodological heterogeneity, and a lack of standardized diagnostic criteria in cases of DPN, CRPS, idiopathic frozen shoulder, OA, and sciatica [20,22,31,33,45]. Taken together, the AMSTAR-2 assessment revealed that the overall methodological quality of systematic reviews investigating immune biomarkers was limited, with most reviews rated as low or critically low confidence.

Besides inflammatory biomarkers, research on proteomic biomarkers might also be significant for chronic pain. Chronic pain requires a multi-dimensional biological signature. Inflammatory biomarkers and a panel of proteins, including transferrin, α2-macroglobulin, may be used in combination with machine learning [50], thereby providing a more accurate diagnosis. However, exploration of broader immune and proteomic markers (e.g., macrophage migration inhibitory factor (MIF) and fecal glyceraldehyde) is not validated yet [26,29]. The AMSTAR-2 assessment also revealed that these systematic reviews were of low quality.

The next identified biomarker was neuroimaging. The recent evidence may prove that reduction in gray matter in the insula [28], which is responsible for interception and emotion, suggests that chronic pain may “rewire” the brain and create a self-perpetuating cycle that integrates the physical sensation with psychological distress. This could reflect fundamental neuroplastic change triggered in patients with prolonged pain. This may also be proved by EEG, where anatomical shift in GBOs suggests that chronic pain is fundamentally different from acute pain [59], most likely due to the role of cognitive and emotional factors in the maintenance of chronic pain. On the other hand, such neuroimaging as MRI fails to provide any data on the patient’s reported symptoms (e.g., OA) [42]. This probably shows that chronic pain is a complex phenomenon that is not only affected by central sensitization but also by a range of factors. AMSTAR-2 indicated that the evidence base for neuroimaging biomarkers was methodologically limited.

Another biomarker, CGRP, has shown promise in several pain conditions: headache, cluster headache, and headache attacks [55,61]. CGRP might be a target biomarker for therapies, although other genetic and neurochemical biomarkers (beta-endorphin, OPRM1, and COMT genes) show conflicting evidence [39]. Such biomarkers as opioid system receptors and substance P (proven working on animals) may guide the design for future human trials [18]. Nevertheless, AMSTAR-2 showed that the systematic review was evaluated as of low quality.

Hormonal biomarkers, although only emerging, reflect the complexity of chronic pain. The use of non-invasive fluid, like saliva (salivary cortisol biomarker), may help to avoid the confounding stress of invasive procedures and encourage the creation of multi-systemic models of pain prognosis [49,53]. Since the studies about hormonal biomarkers are recent, other hormonal markers like HPA, metabolites, or cortisol in neonates have less conclusive results [24].

Regarding joint (CTX-II and serum CRP, and COMP), skin (nerve fiber density), and other tissue-specific biomarkers (CPM), current evidence may not recommend them as reliable biomarkers [20,30,41,44,46].

### 4.1. Gaps and Future Directions

One of the major gaps of the included studies was the lack of high-quality data. Most of the studies were deemed to be of low quality due to the scarcity of longitudinal studies. The included studies in systematic reviews were cross-sectional, and the identification of a causal relationship between biomarkers and pain intensity could not be performed. Next, there has been methodological heterogeneity. Studies on the same pain condition or disease used different assays, collection time points, and pain assessment scales. Thus, the reliability of meta-analyses was hindered by a lack of standardization protocols. Furthermore, many studies were limited by small sample sizes, making it difficult to generalize the findings. The accumulation of consistent evidence-based findings may be achieved if future studies focus on longitudinal studies, protocol standardization, and inclusion of broad and diverse cohorts.

The findings of this umbrella review pose an important distinction between the research value and the clinical value of pain-related biomarkers. From a research perspective, the identified biomarkers provide meaningful insights into the pathophysiology of chronic pain, supporting contemporary models that emphasize neuroplasticity, immune activation, and central sensitization. Biomarkers serve primarily as tools for hypothesis generation, mechanistic exploration, and patient phenotyping in experimental settings. The clinical value of these biomarkers remains limited. Current evidence does not support the routine clinical implementation of pain biomarkers, either as diagnostic tools or as guides for personalized therapy. The translation of biomarker utility into a clinical reality requires a large-scale, valid, and mechanism-based approach. If current methodological limitations are addressed, next step is prioritizing multi-biomarker panels. Combining different biomarker types to develop a multidimensional panel may be used for predicting pain comprehensively (involving various pain phenotypes). Additionally, focusing on non-invasive biomarkers, such as saliva, urine, or EEG, may facilitate their adoption in routine clinical settings.

### 4.2. Limitations of the Umbrella Review

One of the limitations of this umbrella review is that it included only articles published in English because resources for the translation of articles in other languages were not available. This may have introduced language bias. The next limitation is that the AMSTAR-2 assessment revealed that many of the systematic reviews were of low or critically low quality, with methodological flaws such as insufficient justification for study design, incomplete reporting of excluded studies, and inconsistent risk of bias assessment. Since the strength of evidence is constrained by the quality of the systematic reviews, the findings must be interpreted with due caution. In addition, the scope of several reviews was narrow, frequently focusing on specific biomarkers or single pain conditions, which further limits the generalizability of the synthesized findings. Moreover, categorization of evidence strength was based on the conclusions drawn by the included studies, as summarized in Table 1, rather than on an independent certainty assessment, and should be interpreted as indicative rather than definitive. Finally, no formal assessment of overlap was conducted. This may have led to partial duplication and should be considered when interpreting the results.

## 5. Conclusions

The synthesis of evidence from systematic reviews and meta-analyses concludes that no single biomarker for chronic pain has achieved sufficient prospective validation for routine clinical use. Nevertheless, the evidence highlights several promising biomarker domains: inflammatory biomarkers (CRP, IL-6, TNF-α) that are responsible for inflammatory pain phenotype, neuroimaging and neurophysiological biomarkers that indicate central neuroplastic changes, and multi-protein panels that may exhibit high diagnostic accuracy.

In summary, the current evidence advances understanding of the biological and neurophysiological mechanisms underlying chronic pain, rather than providing tools ready for clinical decision-making. The translation of biomarker research into clinical practice remains limited by methodological heterogeneity, cross-sectional designs, and the lack of longitudinal and outcome-driven validation. Next steps involve addressing the pervasive methodological limitations via large, standardized, and longitudinal studies to reduce the gap between research area and clinical practice.

## Figures and Tables

**Table 1:** Characteristics of included studies.

Citation	Study Design (SR&MA of RCTs or Observational Studies?)	Goals of the SR (Very Briefly)	Diseases/Conditions (in Which Biomarkers Were Studied)	Brief Characteristics of Patients	Biomarkers (Including Clinical, Biochemical, Radiological, etc.)	Number of Patients Included in the SR	Total Number of Studies Included in the SR	Effect of Biomarkers on Decision-Making and Outcomes (Were They Useful in Diagnosis, Prescription of Treatment and Improvement of Outcomes)	Benefits of Studied Biomarkers	Limitation of Studied Biomarkers	Future Directions and Opportunities (Both Research and Clinical)	Study Conclusions/Comments
Zebhauser 2023, Germany [15]	SR of observational studies + semiquantitative analysis	Resting-state EEG/MEG as biomarkers for chronic pain	Trigeminal neuropathy, chronic pancreatitis, post-herpetic neuralgia, SCI, breast cancer, MS, failed back surgery, CINP, fibromyalgia, somatoform pain disorder, LBP, orofacial pain, OA, endometriosis, SCD, ankylosing spondylitis, IBD	Adults	Resting-state EEG and MEG	4094	76	Cross-sectional studies: θ and β power may serve as diagnostic biomarkers. Longitudinal and descriptive studies: no clear utility for monitoring or predictive biomarkers	Non-invasive and scalable	Heterogeneity	Differentiating biomarker types: diagnostic vs. monitoring vs. predictive. Including other neuropsychiatric disorders. Standardized protocols.	θ and β power may serve as diagnostic biomarkers
Sanabria-Mazo 2022, Spain [16]	SR of observational studies	To investigate immune-inflammatory and HPA axis biomarkers	Non-specific LBP	Mean age: 21–75 Germany, US, Canada, Australia, Brazil, Norway, Israel, China	Inflammatory: IL-6, IL-10, IL-17, IL-23, IL-1β, TNF-α, sTNF-R1, TGF-β, IFN-γ, GDF-15, CRP, hsCRP. HPA axis: cortisol.	4808	14	Not clearly useful for diagnosis or treatment decisions.	GDF-15, IL-23, sTNF-R1 increased in non-specific LBP	Small sample sizes, heterogeneity	Clarify direction of cortisol response.	Limited evidence for alterations in inflammatory biomarkers and cortisol dysregulation
Gomez-Pilar 2022, Spain [17]	SR of observational studies	Functional activity biomarkers from EEG, MEG, fMRI, and PET that can differentiate between chronic (CM) and episodic migraine (EM)	Chronic and episodic migraine	Adults	EEG, MEG, fMRI, PET	Not all studies reported sample size	24 (22 original articles, 2 review articles)	Differentiate CM vs. EM beyond just number of headache d/mo. Could help guide personalized treatment.	Consistent functional differences between CM and EM	No single reliable, accurate brain activity biomarker	Focus on beta bands for M/EEG studies. Focus on pain/emotion circuits for fMRI/PET studies.	Consistent differences between CM and EM
Matesanz-García 2022, Spain [18]	SR of animal studies	The effects of physiotherapeutic interventions on biomarkers of neuropathic pain	Peripheral neuropathic pain models: traumatic nerve injury, DPN, CINP	Animal models	Immune (cytokines, chemokines, immune cell markers), neurotrophins (NGF, BDNF), opioid system (endorphins, receptors), neurotransmitters (substance P), ion channels (TRPV1).	Rats and mice	85	N/A	Provide insights into MoAs of physiotherapeutic interventions on neuropathic pain. May guide design of future clinical trials.	High RoB in studies. Heterogeneity of biomarkers and methods. Mostly male animals	Studies in both sexes needed. Clinical trials needed to translate findings to humans.	Physiotherapeutic interventions modulate biomarkers of neuropathic pain in preclinical models, particularly neuro-immune biomarkers.
Mussigmann 2022, France [19]	SR of observational studies	EEG biomarkers of chronic neuropathic pain	Chronic neuropathic pain (central or peripheral)	Mean age: 35–64	Resting-state EEG	241	14	EEG biomarkers could help make diagnosis of neuropathic pain more objective and predict response to treatments	Non-invasive, widely available, and provides high temporal resolution	Small heterogeneous studies	Larger studies. Advanced EEG techniques: source localization, connectivity, and machine learning for biomarker identification	Consistent resting-state EEG changes occur in chronic neuropathic pain patients, notably increased θ power and decreased α/β power.
Andronic 2022, Switzerland [20]	SR + MA of observational studies	To summarize the current evidence on skin biomarkers in CRPS Type I	CRPS Type I	N/A	Skin morphological (skin thickness, nerve fiber density, mast cell expression, receptor expression), inflammatory (ILs, TNF-α), vascular (ET-1, NOx), metabolic (lactate), and neuropathic (neurite loss).	299	11	Biomarkers provided insights into pathological processes but did not directly influence diagnosis or treatment decisions.	N/A	Lack of standardized diagnostic criteria. Limited sample sizes	Larger longitudinal studies. Studies on biomarker-guided treatments.	Limited evidence
Kumbhare 2022, Canada [21]	SR + MA of observational studies	Blood biomarkers’ levels in patients with fibromyalgia	Fibromyalgia	Age: 24–61 Mostly women	Cytokines (IL-1β, IL-6, IL-8, TNF-α), CRP, BDNF, IGF, and GH.	57,105	54	Differences in levels of IL-6, IL-8, TNF-α, and BDNF between fibromyalgia patients and controls. No biomarker was diagnostic or specific for fibromyalgia. Potential value of a panel of biomarkers for identifying pathologies or phenotypes.	Potential to identify central sensitization, fatigue, and sleep disorders commonly associated with fibromyalgia. May help stratify patients into subgroups.	Heterogeneity	Large multicenter trials, identifying underlying pathologies, patient subgroups and treatment responses. Develop standard protocols.	Evidence does not support blood biomarkers as specific diagnostic tests for fibromyalgia
Baka 2021, Germany [22]	SR + MA of observational studies	Association of painful DPN with a specific inflammatory profile.	T2DM neuropathy, 1 study—type 1 Mean age: 50–60	Cytokines (TNF-α, IL-2, IL-6), chemokines, growth factors	Systemic inflammatory	3469	13	TNF-α differentiates painful from not painful DPN. Inflammatory markers predict DPN development.	Identify role of inflammation in DPN pathophysiology.	Heterogeneity, study design	Longitudinal and interventional anti-inflammatory drug studies. Larger samples (>150 per arm).	Specific inflammatory profiles differ between painful and not painful DPN.
Bonifácio de Assis 2021, Brazil [23]	Scoping review	Evidence on beta-endorphin as a biomarker for chronic pain treatment with non-invasive brain stimulation	Chronic LBP, PLP, migraine, fibromyalgia, knee OA	Age: 20–77 Mostly female	Beta-endorphin	350	6	Low baseline beta endorphin may predict greater pain intensity	May be used for various chronic pain conditions	Small sample sizes, limited follow-up times, lack of control groups	Research on motor cortex stimulation and longer follow-up; BE as diagnostic and treatment monitoring tool	Limited evidence
Aroke & Powell-Roach, 2020, USA [24]	SR of observational studies	Potential metabolomic signatures associated with chronic pain conditions	Fibromyalgia, OA, migraine, MSK pain, RA, chronic LBP, neuropathic and nociceptive pain, interstitial cystitis/bladder pain syndrome	Adults USA, Netherlands, Italy	Metabolomics biomarkers: amino acids, lipids, carbohydrates	16,876	18	Potential to identify diagnostic and prognostic biomarkers, but none validated for clinical use yet.	Insights into metabolic pathways involved in chronic pain	Heterogeneity, study design	Large-scale studies needed. Standardized measures of pain. Compare invasive and non-invasive samples.	Alterations in metabolites are associated with chronic pain conditions, suggesting involvement of protein, lipid, carbohydrate metabolic pathways.
Morris 2020, Canada [25]	SR + MA of observational studies	Association between inflammatory biomarkers, clinical presentation, and outcomes in patients with non-specific LBP	Non-specific LBP	Adults	CRP, IL-6, TNF-α, IL-1β	384	7	Limited evidence, no clear effect	N/A	Heterogeneity	Longitudinal studies evaluating range of biomarkers and outcomes	Very low to low level evidence for association between studied biomarkers and NSLBP.
Vadasz 2020, international [26]	SR of observational studies	Compare clinical features and biomarkers between MPS and DOMS	MPS, DOMS	Adults	Clinical features (muscle tenderness, decreased RoM), cytokines (TNF-α, IL-1β, IL-6), GFs (FGF-2, PDGF), extracellular matrix proteins (MMP-9), markers of ATP metabolism and hormones (CK, cortisol)	N/A	53	Potentially useful for diagnosis of MPS in future	Insights into pathophysiology and potentially useful for diagnosis of MPS in future	Heterogeneous	Validate proposed biomarkers for MPS diagnosis; assess diagnostic performance of biomarkers; establish reproducibility and generalizability	MPS and DOMS share clinical and biomarker similarities
Lim 2020, Australia [27]	SR + MA of observational studies	To investigate whether inflammatory biomarkers are associated with nonspecific LBP	Non-specific LBP	Age: 19–71 Males and females	CRP, hsCRP, TNF-α, TNF, sTNF-R1, IL-6, IL-1β, fibrinogen	15,842	13	N/A	Elevated inflammatory biomarkers, particularly CRP, TNFs and IL-6, are associated with nonspecific LBP. Inflammation role in the pathogenesis	Heterogeneity	Longitudinal studies	Elevated inflammatory biomarkers, particularly CRP, TNFs and IL-6, are associated with nonspecific LBP.
Henssen 2019, Netherlands [28]	SR + MA of observational studies	Identify concordant structural and functional brain changes in trigeminal neuralgia	Trigeminal neuralgia	Age: 54–56 Both genders	Structural MRI (gray matter density changes), functional MRI (functional connectivity)	381	11	Changes seen in thalamus, cingulate, insula. Provide insights into pathophysiology	Identify involved brain regions to understand mechanisms and pathology.	Single subject inference not possible. Functional significance of changes not fully clear.	Focus imaging on identified regions. See if changes normalize after treatment. Develop as diagnostic biomarkers.	Common structural and functional changes found which elucidate mechanisms in trigeminal neuralgia. Results as biomarkers for diagnosis/treatment response.
Magalhaes 2019, Brazil [29]	SR + MA of observational studies	To investigate possible biomarkers for the diagnosis and symptom evaluation of BPS	BPS	Age: 40–60 Mostly females	Urine (MIF, NGF, Etio-S, methylhistamine, histamine, IL-6, APF, EGF, HB-EGF, G5P1, chemokine profile, DNA methylation), stool (microbiome, glyceraldehyde), bladder biopsy (gene expression, nerve density, cytokines)	643	11	No clear evidence	Clues to pathophysiological mechanisms in BPS	No validation, lack of correlation with symptoms	Prospective validation studies needed correlating biomarkers with symptoms and treatment response	Urinary MIF, NGF, Etio-S, APF, and methylhistamine/IL-6, fecal glyceraldehyde, bladder epithelial expression of genes were increased. Urinary DNA methylation in CpG sites, MCP-3, G5P1, and HB-EGF levels, CHT, HB-EGF, OCT-1, SMRT-1, WNT11 expression were reduced
Fernandes 2019, Spain [30]	SR + MA of observational studies	Validity of CPM as a biomarker of chronic pain by correlation with clinical pain outcomes (intensity, disability, duration, number of areas)	Knee OA, back pain, fibromyalgia, temporomandibular disorders, IBS	Mean age: 50.1 61% females	CPM	1958	32	CPM was not found to strongly correlate with clinical manifestations of pain	CPM is easy to obtain	Heterogeneity	Standardized CPM protocols	The review does not support CPM as a valid biomarker of clinical pain
Andronic 2020, Switzerland [31]	SR + MA of observational studies	Evidence on biomarkers related to pathophysiology of idiopathic frozen shoulder and underline their clinical implications	Idiopathic frozen shoulder	N/A	Cytokines (ILs, TNF-α, COX-1/2), MMP/TIMP, TGF-β1, tenascin C, ASIC1/3, vimentin, collagen types, β-catenin, IGF-2, melatonin receptors	333	15	Biomarkers provided insights into pathophysiology but unclear effect on diagnosis or outcomes.	Identified roles of inflammation, extracellular matrix remodeling, neurogenesis, and fibrosis.	Lack of disease staging, previous injections/surgeries may confound results.	Studies should stage disease, exclude previous treatments, evaluate clinical utility of findings.	Abnormal neurogenesis, extracellular matrix changes, inflammation, impaired healing, and fibrosis interact in frozen shoulder pathophysiology.
Ping 2019, China [32]	SR of reviews, human and animal studies	To synthesize the literature on the central sensitization mechanism of endometriosis-associated pain	Endometriosis	Mean age: 32.7–43.3	BDNF, TNF-α, NO, NK1R gene polymorphism rs881, TRPV1, substance P, CGRP	509	15 (3 animal studies)	Biomarkers helped understand the pathogenesis and mechanisms of endometriosis pain. Not enough evidence to guide clinical decision-making and treatment.	Provided insights into central sensitization mechanism of endometriosis pain.	Limited sample sizes, lack of RCTs	Larger RCTs	Endometriosis pain involves central sensitization.
Jungen 2019, Netherlands [33]	SR + MA of observational studies	Evaluate the role of inflammation in sciatica by examining inflammatory biomarkers and their association with clinical symptoms	Sciatica (mostly chronic)	Mean age: 26–52	Cytokines (ILs, TNF-α), CRP, chemokines, phospholipase A2 measured in serum, CSF, disk biopsies	1212	16	Insufficient evidence to recommend anti-inflammatory treatments based on biomarker levels	IL-21, TNF-α, and CRP	Heterogeneity	Study biomarkers in acute sciatica, standardize methods, patient subgroups that may benefit from anti-inflammatory treatments	Insufficient evidence
Gardner 2019, UK [34]	SR + MA of observational studies	To identify and compare clinical markers and biomarkers which predict analgesic response to radiotherapy for cancer-induced bone pain	Cancer-induced bone pain	Adults	Urinary markers of osteoclast activity (pyridinoline, deoxypyridinoline), genetic biomarkers (saliva: SNPs), imaging (FDG-PET, CT, MRI DWI), patient demographics, disease parameters, quantitative sensory testing, physical activity/gait assessment, spinal instability classification	4490	21	No biomarkers were definitively identified that can currently guide clinical decision-making or improve outcomes.	Some potential to identify patients likely to benefit from radiotherapy, avoid unnecessary side effects and hospital visits in non-responders, and better use healthcare resources.	Small sample sizes, heterogeneity	Larger prospective studies needed, with consistent outcome definitions	There is currently no robust clinical marker or biomarker that predicts response to radiotherapy for cancer-induced bone pain
Teraguchi 2018, China, Hong Kong [35]	SR of observational studies	To assess association between HIZs on lumbar spine MRI and LBP	LBP	Mean age: 21–50	MRI HIZs	1541	6	Unclear	Potential imaging biomarker for discogenic LBP	Small sample sizes, heterogeneity, lack of standardized imaging protocols	Large-scale studies with standardized imaging/phenotyping	Evidence HIZs may be risk factor for LBP
Van Den Berg 2018, Netherlands [36]	SR + MA of observational studies	Association between pro-inflammatory biomarkers and the presence and severity of nonspecific LBP	Non-specific LBP	Mean age: 30–76	Pro-inflammatory: CRP, IL-6, TNF-α, RANTES	16,346	10	Unclear, biomarkers not directly used for diagnosis/treatment decisions	Association between CRP, IL-6, TNF-α and presence/severity of non-specific LBP	Heterogeneity, lack of longitudinal data; potential confounding	Longitudinal studies, identify subgroups and guide treatment.	Association between pro-inflammatory biomarkers and presence/severity of non-specific LBP
Andrade 2018, Brazil [37]	SR of non-RCT	To identify the acute effects of physical exercise on inflammatory biomarkers in patients with fibromyalgia	Fibromyalgia	All females Mean age: 38.9–54	Pro-inflammatory cytokines (IL-6, IL-8, IL-1β, IL-18, TNF-α), anti-inflammatory cytokine (IL-10), stress protein (Hsp72)	238	6	Unclear	Provide information about inflammatory status and response to exercise in fibromyalgia patients	Small sample sizes, heterogeneity	RCTs with larger sample sizes, standardized protocols and time points	Fibromyalgia patients may have chronic low-grade inflammation at baseline based on altered biomarker levels.
Wallwork 2017, Australia [38]	SR + MA of observational studies	To evaluate defensive reflex parameters (threshold, size, latency, duration) in people with and without clinical pain	Migraine, tension headache, fibromyalgia, RA, back pain, whiplash, IBS, lateral epicondylalgia.	Mean age: mid-20s–60 Mostly females	Defensive reflexes: blink reflex, nociceptive flexion reflex, startle response. Parameters: threshold, size, latency, and duration.	2223	17	N/A	Lower reflex thresholds were found in pain groups compared to controls	Heterogeneity, missing data	More data	Activation thresholds of defensive reflexes are lower in chronic pain patients vs. controls. This lowered threshold was not confined to the painful body part, suggesting a central rather than peripheral mechanism. The reflex upregulation may reflect increased need for bodily protection rather than sensitization.
Bjorland 2016, Norway [39]	SR + MA of observational studies	Genetic factors and biomarkers predicting pain recovery in patients with newly diagnosed lumbar radicular pain.	Lumbar radicular pain caused by lumbar disk herniation	Mean age: 41–47	Genetic variants (OPRM1, COMT, MMP1, IL-1α, IL1-β, IL-6, GCH1); proteins (IL-1 β, IL-6, TNF-α, IFN-α, hsCRP, CGRP1, Galanin, Neuropeptide 4, Substance P).	872	15	Limited evidence	Potentially useful prognostic biomarkers	Heterogeneity	Larger cohorts, combined analyses of genetic, biomarker and clinical data.	Several genetic factors and biomarkers may be linked to poor recovery in lumbar radicular pain
Gold 2016, Sweden [40]	SR + MA of observational studies	Quantitative imaging biomarkers associated with neck and shoulder	Rotator cuff tears, adhesive capsulitis, shoulder impingement, trapezius myalgia, neck/shoulder pain	Mean age: 22–50	Imaging: MRI, US, infrared thermography, NIRS. Metrics: muscle size, vascularity, O_2_ saturation.	4781	49	Biomarkers may help with diagnosis and disease monitoring	May detect subclinical disease, disease progression	Study quality, heterogeneity	Longitudinal studies, biomarker changes with disease onset and progression	Limited evidence for some quantitative imaging biomarkers like reduced neck muscle size in neck pain and reduced trapezius muscle oxygenation in myalgia.
Dell’Isola 2016, Scotland [41]	SR + qualitative synthesis of observational studies	To identify evidence for distinct clinical phenotypes in knee OA	Knee OA	Adults	Clinical variables: pain, psychological and metabolic factors, imaging, genetics, biomechanics.	Not reported in some studies	25	Biomarkers may identify subgroups that could benefit from targeted/personalized treatments.	May allow identification of distinct clinical phenotypes based on different underlying disease mechanisms.	Lack of standard phenotype definitions, heterogeneity, potential overlap between phenotypes.	Define and confirm phenotypes. Assess overlap between phenotypes. Validate ability of biomarkers to predict phenotype and treatment response.	6 potential clinical phenotypes in knee OA: chronic pain, inflammatory, metabolic syndrome, bone/cartilage metabolism, mechanical overload, minimal joint disease.
Akinci 2016, international [42]	Narrative review	Guide on evidence for central sensitization in chronic OA pain and how to address it clinically.	OA with central sensitization	Adults	Nociceptive withdrawal reflexes, quantitative sensory testing, cortical event-related potentials, functional MRI, magnetic source imaging.	N/A	N/A	Biomarkers may help confirm central sensitization when there is diagnostic uncertainty.	Evidence for central sensitization.	Lack of validation and availability.	Confirm suitability of biomarkers for clinical use.	Central sensitization is common in a subgroup of OA.
Kawi 2016, USA [43]	SR + MA of observational studies and RCTs	Studies using exercise interventions in chronic MSK pain that measured biomarkers in pain pathways, describe the effects of exercise on these biomarkers, and evaluate associations between biomarkers and pain-related outcomes.	Chronic LBP, ankylosing spondylitis, knee OA, fibromyalgia, chronic fatigue syndrome	Adults Mostly females Mean age: 30–70	Inflammatory (IL-6, IL-8, IL-1β, TNF-α), neurotransmitters (COMT, adrenergic receptors), metabolite-detecting (ASIC3, P2X4, P2X5, TRPV1)	1087	12	Exercise appeared to influence levels of some biomarkers like cytokines and neurotransmitters. Certain biomarkers like COMT were associated with decreased pain, disability, anxiety, depression after intervention	May provide insight into mechanisms and individual variability in chronic pain.	Inconsistent findings, heterogeneity	Larger samples, replication, optimal exercise dose, longitudinal studies	Exercise impacts some biomarkers in pain pathways
Nepple 2015, USA [44]	SR of observational studies	To assess molecular biomarkers in the pathophysiology of hip OA, diagnosis, disease staging, and prognosis	Hip OA	Mean age: 56–75 US, Europe, Japan	Urinary CTX-II, serum CRP, COMP, Helix-II	Difficult to assess–reported several times	40	No biomarker has been adequately validated for clinical use yet.	Promise hip OA diagnosis, staging and prognosis.	Heterogeneity	Validate urinary CTX-II, serum CRP and COMP. Prearthritic conditions. Develop biomarkers specific to hip joint.	Molecular biomarkers have potential for diagnosis, staging and prognosis of hip OA.
Jin 2015, Australia [45]	SR + MA of observational studies	To determine if CRP levels are elevated in OA, and correlate CRP levels with radiographic changes and symptoms.	OA	Mean age: 61.4 52% females	hs-CRP	17,090	32	Associated with pain and physical dysfunction in OA, but not with radiographic changes. Suggests systemic inflammation more related to symptoms than structural damage in OA.	Easy, standardized blood test that provides a systemic marker of inflammation.	Heterogeneity between studies. No causal evidence from longitudinal studies. Confounding by obesity.	Longitudinal studies adjusting for confounders like BMI	Low-grade inflammation may play a greater role in symptoms than structural changes in OA
Hunter 2011, Australia, UK, USA [46]	SR + MA of observational studies	Summarize literature on concurrent and predictive validity of MRI biomarkers in OA	OA (knee, hip, hand)	Mean age: 12.6–74.8 Majority—females	MRI—quantitative cartilage morphometry, compositional MRI techniques, semi-quantitative assessment of cartilage, synovium, bone marrow lesions, meniscus, ligament	18,346	142	Cartilage volume change and presence of defects/lesions predicted TKR. Cartilage loss weakly predicted symptom change.	Visualization of multiple joint tissues affected in OA. Assessing effectiveness of interventions. Cartilage volume change and defects/lesions can predict outcomes.	Inconsistent relation to symptoms. Weak correlation with radiographic change. Lack of a gold standard outcome measure in OA.	Improve predictive validity of MRI biomarkers for important clinical outcomes. Develop MRI biomarkers more closely related to symptoms.	MRI has advantages in visualizing individual joint pathologies in OA and predicting some clinical outcomes.
Mauri, 2025, Italy [47]	SR and MA	Analyze the effects of different types of exercise interventions (aerobic, resistance, combined, neuromuscular, etc.) on pain, functional capacity, and inflammatory biomarkers in people with osteoarthritis	Osteoarthritis (OA) (mostly knee OA)	Aged between 38 and 85 years;	-TNF receptor 1 and 2 (sTNFR1; sTNFR2) -Serum C-reactive protein (CRP) -Interleukin-6 (IL-6) -Interleukin-1 beta (IL-1β) -Interleukin-10 (IL-10) -Interleukin-8 (IL-8) -Tumor necrosis factor alpha (TNF-α) -Serum cartilage oligomeric matrix protein (COMP) -C-terminal cross-linked telopeptide of type II collagen (CTX-II) -Plasma matrix metalloproteinase 1 and 3 (MMP-1; MMP-3) -Resistin (RSTN)	N1461	21; 11 in the meta-analysis	Resistance training and neuromuscular training reduce key inflammatory markers such as CRP, IL-6, and TNF-α, which are associated with OA-related inflammation.	Understanding pathogenesis and pain mechanisms of OA; Monitoring disease progression and intervention effectiveness; Identifying effective interventions;	Heterogeneity of study designs and assessments; Limited number of studies; No clear consensus exists on the most effective biomarkers to track OA progression or response to exercise interventions; Risk of bias in selective reporting and incomplete outcome data domains; Potential confounding (since many individuals had sedentary lifestyle at baseline)	More rigorous RCTs with better controls and standardized protocols; Focus on the effects of neuromuscular and Resistance Training on inflammatory biomarkers; Investigate interrelationships between pain relief, inflammatory biomarker modulation, and improvements in functional assessments	Neuromuscular training is highlighted as a key intervention, most effective (in terms of pain reduction in knee OA patients); Resistance and combined training also were efficient at improving pain outcomes and functionality/
Lozano-Parra, 2024 Colombia [48]	SR	To evaluate the potential role of immunological biomarkers in predicting the progression to persistent or chronic joint pain (arthralgia) after the acute phase of the disease in patients with Chikungunya virus	Chikungunya virus infection (CHIKV)	Age range of 0 to 90 y.o.; Geographic coverage of studies: Asia (52.6%), America (31.6%), Europe (13.2%), and Africa (2.6%	-Interleukin-6 (IL-6); -Tumor necrosis factor-alpha (TNF-α); -Interferon-gamma (IFN-γ); -Interleukin-8 (IL-8); -Interleukin-10 (IL-10);	Individual study sample sizes range from 8 to 346 participants;	38	Inflammatory response during the Acute phase—elevated proinflammatory cytokines (IFN-α, IFN-γ, IL-2R, IL-6, IL-7, IL-8), anti-inflammatory cytokines (IL-1Ra, IL-4), chemokines (MCP-1, MIG, IP-10), and growth factors (VEGF, G-CSF); IL-6, IL-4, immunoglobulin G (IgG) levels, and C-reactive protein (CRP)—potential prognostic biomarkers indicating the risk of developing chronic arthritis or persistent joint pain after CHIKV infection. However, no unequivocal set of biomarkers can reliably predict progression to chronic arthropathy or guide clinical decision-making	Improve understanding of disease pathogenesis; IL-6, IL-4, IgG antibodies, and CRP, —potential prognostic indicators that may predict the risk of developing persistent joint pain or chronic arthritis after acute infection. It could help early risk stratification of patients.	The heterogeneity among studies in terms of populations, biomarker measurement timing, and outcome definitions; Meta-analyses or pooled quantitative syntheses were not applicable, unable to draw robust conclusions High risk of bias; No adjustment for relevant covariates;	Standardized definitions for chronic arthropathy outcomes, uniform measurement timings, and larger, prospectively designed cohorts to improve data consistency; Longitudinal designs for identifying the set of reliable prognostic immunological biomarkers; Understand the mechanisms of the change in biomarkers.	Acute-phase immunologic profile in CHIKV infection characterized by elevated pro- and anti-inflammatory cytokines, chemokines, and growth factors.
Rodrigues, 2024 Brazil [49]	SR	To determine changes in salivary biomarkers related to pain, anxiety, stress, and inflammation that occur with tooth movement during orthodontic treatment in children and adolescents.	Patients who need orthodontic interventions	Age range: 8–18 y.o. Predominantly male patients	Immunoglobulins: IgA (in three studies), IgG, IgM, IgD, and IgE; Hormones: Cortisol, leptin; Electrolytes: Calcium (Ca^2+^), phosphate (Pi^3−^), sodium (Na^+^), chloride (Cl^−^), and potassium (K^+^); Enzymes: Alkaline phosphatase (ALP), lactate dehydrogenase (LDH), matrix metalloproteinases 8 and 9 (MMP8, MMP9); Mediators: Soluble receptor activator of nuclear factor Kappa B ligand (sRANKL), osteoprotegerin (OPG), interleukin-1β (IL-1β), prostaglandin E2 (PGE2), bone morphogenetic protein 4 (BMP4)	249	12	The data was scarce, certainty was low to draw firm conclusions	Salivary biomarkers can provide data on patients’ general health status; Biomarkers like enzymes (ALP, LDH, MMP8, MMP9) and mediators (sRANKL, OPG, IL-1β, PGE2) reflect inflammation, bone remodeling, and tissue response during orthodontic tooth movement;	Methodological heterogeneity across included studies; Limited number of studies; Low certainty, small sample sizes.	More high-quality, standardized studies with larger sample sizes and better methodological consistency; Monitoring salivary IgA and assessing oral health-related quality of life;	Orthodontic tooth movement has insignificant to no effect on endogenous salivary biomarkers; Salivary biomarkers hold potential for monitoring and predicting orthodontic treatment stages and adverse effects.
Gkouvi, 2024 Greece [50]	SR	To summarize the proteome of adult patients with fibromyalgia syndrome (FMS) to better understand the disease’s pathophysiology, identify diagnostic and prognostic protein biomarkers.	Fibromyalgia syndrome (FMS).	Mean age range: early 40s to early 50s; Predominantly females; Reported severe pain.	Transferrin (TRFE), Fibrinogen α chain (FGA), Fibrinogen β chain (FGB), Fibrinogen γ chain (FGG), Profilin-1 (PFN1), Complement C4-A (C4A), Complement C1qC (C1qC), Serum amyloid A4 (SAA4); Serum amyloid p-component (SAP); Thrombospondin-1 (THBS1); α2-macroglobulin (A2M); Haptoglobin (Hp); Phosphoglycerate mutase 1 (PGAM1); Transaldolase (TALDO); Calgranulin A (S100-A8); Calgranulin C (S100-A12); Apolipoprotein-C3 (ApoC3); Immunoglobulin fractions (various Ig lambda and kappa chain regions).	242	10	Transferrin and α2-macroglobulin showed moderate association with pain severity; Histidine protein methyltransferase 1 homolog (HMPT1), Interleukin-1 receptor accessory protein (IL1RAP), and Ig lambda chain V-IV region (IGL3-25), achieved diagnostic accuracy up to 0.97 and helped to differentiate FMS patients from controls.	High diagnostic accuracy of HMPT1, IL1RAP, IGL3-25 when combined in decision tree models; Transferrin (TRFE) and α2-macroglobulin (A2M) = indicators of disease and pain severity; α2-macroglobulin, involved in coagulation, inflammation, and autoimmunity, is promising for treating neuropathic pain and osteoarthritis.	Overlapping data across studies; Heterogeneity in proteomics methodologies across studies; Lack of quantitative data and standardized approaches made it impossible to conduct meta-analysis.	More primary studies with standardized methodologies are needed; Studies combining multiple biomarkers to improve diagnostic accuracy and disease phenotyping are needed; Further research should focus on determining if specific protein patterns can predict clinical profiles or treatment responses; the effect of medications and other confounding variables on the proteome; the role of neuroinflammation and its biomarkers in FMS pathogenesis.	Dysregulation of proteins involved in the complement and coagulation cascades, immune system, iron metabolism, and oxidative stress pathways appears to be characteristic of FMS; No validated biomarker panel exists for routine clinical use.
Sima, 2024 Australia, China [51]	SR and MA	To identify which inflammatory biomarkers are associated with back pain, leg pain, and disability.	Low back disorder (LBD), defined as low back pain (LBP) without specific spinal pathologies. Conditions such as spondylolisthesis, spondylosis, disk herniation, disk degeneration, scoliosis deformity, radicular syndromes, and also failed back surgery syndrome.	Mean age = 51 y.o.; 53.2% female (489 Males/653 Females)	IL-1 beta; IL-2; IL-4; IL-6; IL-7; IL-8; IL-10; IL-17; TNF-alpha (TNFα); MCP-1 (Monocyte chemoattractant protein-1); GM-CSF (Granulocyte-macrophage colony-stimulating factor); CXCL6; CXCL12; hsCRP (high sensitivity C-reactive protein); Beta-endorphin; CTX-1 (C-terminal telopeptide of type I collagen);	1142	20	Inflammatory biomarkers were significantly associated with low back disorder (LBD) and clinical outcomes such as back pain, leg pain, and disability scores, but evidence is still weak to draw firm conclusions.	Increase in CTX-1 and IL-10 and decrease in IL-1 beta after treatment = associated with reduction in back pain scores; MCP-1 positively correlated with low back pain and leg pain; IL-8 was associated with increased disability score; Negative associations were found between hsCRP and low back pain and leg pain, and between IL-6 and leg pain;	Heterogeneity in patient populations, pain and disability scales, treatment protocols, and follow-up times across studies; Lack of standardized inclusion/exclusion criteria and insufficient control of confounding factors such as smoking, exercise, and BMI; The moderate quality of evidence and limited number of randomized controlled trials.	Further research focus on confirming findings related to changes in biomarkers (e.g., CTX-1, IL-10, IL-1 beta) after treatment; Evaluating the magnitude of associations between inflammatory biomarkers and clinical outcomes in LBD; Standardize inclusion and exclusion criteria, demographic status of cohorts, and the pain and disability scales; Better control for confounding factors; Rigorous study methodologies.	Inflammatory biomarkers have significant potential to aid understanding and targeted management of LBD, but further research is warranted.
Puerto Valencia, 2024 Germany [52]	SR	To assess how non-drug treatments like exercise, manual therapies, acupressure, and others can regulate inflammation associated with chronic low back pain (CLBP).	Chronic low back pain (CLBP).	Adults (age > 18); (CLBP) that persists or recurs for more than 3 months;	Pro-inflammatory cytokines: Tumor necrosis factor-alpha (TNF-α), Interleukin-1 (IL-1), Interleukin-1 beta (IL-1β), Interleukin-2 (IL-2), Interleukin-6 (IL-6), Interleukin-8 (IL-8), Interferon-gamma (IFNγ); Anti-inflammatory cytokines: Interleukin-4 (IL-4), Interleukin-10 (IL-10); C-reactive protein (CRP); Chemokines: interferon-γ-induced protein 10 (IP-10), Chemokine ligand 2 (CCL2), Chemokine ligand 3 (CCL3), Chemokine ligand 4 (CCL4).	Not reported	13	TNF-α, IL-1β, IL-6, and chemokines such as CCL2 and CCL3 are correlated with pain intensity in CLBP.	Changes in biomarkers reflect the physiological effects of non-pharmacologic interventions, offering measurable biological indicators to assess intervention impact: Decreased tumor necrosis factor-alpha (TNF-α) was observed after osteopathic manual treatment (OMT), neuro-emotional technique (NET), and yoga. Decreased interleukin (IL)-1, IL-6, IL-10, and C-reactive protein (CRP) were reported after NET. Increased IL-4 was reported after acupressure. Exercise decreased TNF-α, IL-1β, IL-8, interferon-gamma (IFNγ), and interferon-γ-induced protein 10 (IP-10), SMT (spinal manipulative therapy) decreased IL-6, CRP, and chemokine ligand 3 (CCL3).	Heterogeneity in patient populations and biomarker measurement methods; Limited number of high-quality studies; Limited sample sizes; No data on clinical outcomes post intervention, therefore guidance for clinical use of non-pharmacologic interventions based on biomarker changes is limited.	More randomized controlled trials with larger sample sizes; Standardizing the methods for biomarker selection and measurement; More studies including appropriate control or comparator groups are needed to distinguish intervention effects from confounding factors; Revealing underlying biological mechanisms of the impact of non-pharmacological interventions on changes in biomarkers.	Non-pharmacologic interventions for CLBP generally inhibit inflammatory processes by reducing pro-inflammatory and increasing anti-inflammatory biomarkers, indicating their potential as therapeutic strategies, but further more rigorous research is needed.
He, 2024 China [53]	SR&MA	To assess whether salivary and serum biomarkers can serve indicators of anxiety, stress, and depression, in patients with burning mouth syndrome (BMS).	Burning mouth syndrome (BMS).	Age range: 26.3–81 y.o.; 81.7% females in BMS group, and 75.8% females in the control group.	-Salivary cortisol -Salivary α-amylase -Serum interleukin-6 (IL-6).	1542	12	Salivary cortisol and α-amylase could be biomarkers of anxiety; Serum IL-6 lacked significant differences and clear associations with psychological distress.	Salivary cortisol was found to be a promising biomarker to reflect psychological status (especially anxiety) in BMS, with significantly higher levels in patients versus controls; Salivary α-amylase may also be a potential biomarker, but further research is required.	Heterogeneity in study designs and biomarker measurement methods; Included case–control designs with some moderate risk of bias; Reduced generalizability of findings.	More robust and standardized clinical studies with larger sample sizes; Explore mechanistic relationships between psychological symptoms and biomarker changes in BMS; Investigating additional potential biomarkers and combining biomarker profiles with psychological scales.	Salivary cortisol could be beneficial for detecting psychological disorders (anxiety and depression) in patients with BMS. Further research is needed for solid evidence.
Yang, 2024 China [54]	SR&MA	To identify robust and consistent gray matter (GM) abnormalities (neuroimaging biomarkers) in patients with chronic low back pain (CLBP).	Chronic low back pain (CLBP), including lumbar disk herniation and ankylosing spondylitis with low back pain.	The average age of patients varied by study, with examples including 41.6 years, 50.7 years, 24.21 years.	Regional gray matter (GM) volume alterations.	574	13	Increased GM volumes in the left striatum and left post-central gyrus in CLBP patients. Decreased GM volumes in the left superior frontal gyrus, left cerebellum, right striatum, left insula, and right middle occipital gyrus in CLBP patients compared to healthy controls.	These regional GM volume changes serve as potential neuroimaging biomarkers for pain chronification.	Potential bias due to the reported coordinates rather than raw imaging data; CLBP patients are clinically heterogeneous; Limited number of studies.	Clarify the precise relationship between GM alterations and pain chronification; Larger and more homogenous samples to address clinical heterogeneity; Specific subgroups of CLBP patients, based on distinct clinical characteristics to provide personalized interventions.	Significant gray matter (GM) alterations in patients with CLBP, specifically showing reduced GM in the left superior frontal gyrus, left cerebellum, right striatum, left insula, and right middle occipital gyrus, alongside increased GM in the left striatum and left postcentral gyrus could be potential biomarkers of pain chronification in CLBP.
Søborg, 2024 Denmark [55]	SR	To evaluate and summarize the existing evidence on biomarkers associated with cluster headache.	Cluster headache (CH), including episodic cluster headache (eCH), episodic cluster headache in bout (eCHb), episodic cluster headache in remission (eCHr), and chronic cluster headache (cCH).	832 patients with CH; 872 controls.	Hypothalamic-regulated hormones: cortisol (the most frequently investigated), other pituitary and hypothalamic hormones such as growth hormone, luteinizing hormone, follicle-stimulating hormone, adrenocorticotropic hormone (ACTH). Inflammatory markers: interleukin 1 (IL-1), soluble intercellular adhesion molecule-1 (sICAM-1), soluble vascular cell adhesion molecule-1 (sVCAM-1), sE-selectin, CD markers (CD3+, CD4+, CD8+, CD57+, CD14+), neuron-specific enolase (NSE), and protein S100B. Neuropeptides: calcitonin gene-related peptide (CGRP), hypocretin-1 (orexin A), β-endorphin.	1704	40	Inconsistent evidence regarding the unique biomarker that can definitively distinguish patients with CH from healthy controls or other primary headaches. Cortisol was the most frequently investigated and was elevated in most studies in patients with cluster headache compared to healthy controls. Interleukin-2 (IL-2), was found to increase during active cluster headache bouts. Calcitonin gene-related peptide (CGRP) was consistently found elevated during cluster headache attack.	The biomarkers contributed to substantiating the underlying biological and pathophysiological mechanisms of CH; Some biomarkers could be indicators of disease activity or phase;	Small sample sizes; Lack of healthy control groups or other headache comparators; Possible selection bias, and variation in laboratory methods.	Larger sample sizes and consistent recording of potential confounders (e.g., medication use, smoking); Prospective meta-analyses and subgroup analyses; Focus on imaging, and gene markers.	There is a need for future high-quality studies with improved methodologies and more comprehensive comparator groups to establish clinically useful biomarkers for CH.
Pinto, 2023 Portugal [56]	SR	To determine whether patients with NsLBP show changes in inflammatory biomarkers.	Non-specific low back pain (NsLBP)	Mean age range: 29 to 71 y.o.; The duration of LBP was categorized as acute (<6 weeks) or chronic (≥6 weeks).	C-reactive protein (CRP) and high-sensitive CRP (Hs-CRP); Interleukin-1 (IL-1) and IL-1β; Interleukin-6 (IL-6); Tumor necrosis factor-alpha (TNF-α); Interleukin-10 (IL-10).	14,555	15	Insufficient evidence to associate these biomarker changes with the degree of pain severity or the activity status of LBP. Individuals with NsLBP showed increased systemic levels of classic pro-inflammatory biomarkers: C-reactive protein (CRP) Interleukin-6 (IL-6) Tumor necrosis factor-alpha (TNF-α); Levels of interleukin-10 (IL-10), an anti-inflammatory cytokine, were found to be decreased in NsLBP patients. High-sensitive CRP (Hs-CRP) and IL-1 were not consistently correlated with NsLBP.	Biomarkers like CRP, IL-6, and TNF-α may reflect systemic inflammation associated with NsLBP; Changes in levels of pro-inflammatory biomarkers (e.g., CRP, IL-6) and anti-inflammatory cytokines (e.g., IL-10) could help identify individuals LBP.	Lack of differentiation of confounding factors like medication use and gender effects; Risk of publication bias; Small sample sizes, lack of blinding, inadequate follow-up, or unclear exposure ascertainment.	Use of larger longitudinal cohorts, blood biobanks, and standardized pain assessment methods	There is evidence that individuals with non-specific low back pain exhibit increased systemic pro-inflammatory biomarkers—specifically C-reactive protein (CRP), interleukin 6 (IL-6), and tumor necrosis factor α (TNF-α)—and decreased levels of the anti-inflammatory biomarker interleukin 10 (IL-10), but further research is warranted.
Saravanan, 2023 USA [57]	SR	To identify specific proinflammatory cytokines associated with axial low back pain (aLBP) in adults.	Axial low back pain (aLBP), including chronic low non-radicular pain, disk-related chronic low back pain, and acute back pain.	Predominantly females, with the majority from Norway, followed by the USA 82% of studies included participants aged 35 years and above.	Interleukin-6 (IL-6); Tumor Necrosis Factor-alpha (TNF-α); C-Reactive Protein (CRP); Interleukin-17 (IL-17); Interleukin-1 beta (IL-1β); Interleukin-8 (IL-8); Tumor Necrosis Factor Receptor 1 (TNF-R1).	66,946	11	Biomarkers hold promise for refining diagnosis and guiding personalized interventions in aLBP but require further validation; Key proinflammatory cytokines—C-Reactive Protein (CRP), Tumor Necrosis Factor-alpha (TNF-α), and Interleukin-6 (IL-6)—were consistently associated with pain intensity in adults with aLBP.	Proinflammatory cytokines may serve as composite biomarkers for pain, behavioral symptoms, and comorbidities in aLBP.	No meta-analysis; Did not address behavioral symptoms and comorbidities comprehensively.	Need for more well-designed experimental studies; Inclusion and control for confounding variables such as diet, physical activity, and lifestyle factors.	There is evidence that three proinflammatory cytokines (CRP, TNF-α, and IL-6) are associated with pain intensity in aLBP.
Beiner, 2023 Germany [58]	SR&MA.	To summarize the current evidence on hypothalamic–pituitary–adrenal (HPA) axis and sympathoadrenal (SAM) axis biomarkers—including cortisol, ACTH, CRH, epinephrine, and norepinephrine—in individuals with fibromyalgia syndrome (FMS).	Fibromyalgia syndrome (FMS).	Predominantly females; 70% of the patients were recruited from clinical settings and 30% from nonclinical settings.	HPA-axis hormones: -Corticotropin-releasing hormone (CRH) -Adrenocorticotropic hormone (ACTH) -Cortisol (with subgroups analyzed: blood cortisol, salivary cortisol, urinary cortisol, morning cortisol) SAM-axis hormones: -Epinephrine -Norepinephrine	2657 (1465 individuals with fibromyalgia syndrome (FMS) and 1192 FMS-free controls)	47	No main effect of FMS on altered levels of CRH, ACTH, blood cortisol, morning cortisol, and epinephrine; Biomarkers were not reliably altered in FMS patients and may be influenced more by population-specific or study-specific variables; Decreased salivary and urinary cortisol combined with increased norepinephrine levels may indicate adrenocortical hypofunction with increased sympathetic tone in FMS patients.	The biomarker data suggest that different subtypes of FMS may exist, with differing cortisol and norepinephrine profiles. This could help in distinguishing patient subgroups	High heterogeneity and significant evidence of publication bias.	Clearly distinguish subgroups within FMS patients, as hormonal patterns may vary between these groups; Controlling for time of hormone sampling (due to circadian rhythms), medication, nutrition, and other confounding variables; Mechanism-based longitudinal research.	Due to high heterogeneity, methodological differences between studies, and the lack of clear consistent patterns, biomarkers cannot yet be used reliably for diagnostic decision-making or individualized treatment.
Li, 2023 China, USA [59]	SR&MA;	To analyze and meta-analyze gamma-band oscillations (GBOs) related to pain in both humans and rodents, to characterize their temporal, frequential, and spatial features, and to understand their relationship with different types of pain.	Chronic pain, tonic pain, and neuropathic pain	Not reported	Gamma-band oscillations (GBOs)	Not reported	73 human studies and 17 rodent studies.	Evidence showed significant correlations between GBO magnitude and subjective pain intensity across different pain types and species; GBO could be a promising biomarker for pain perception. Frequency of GBOs and different types of pain: Phasic pain induced higher-frequency GBOs (~66 Hz) mainly localized to the sensorimotor cortex, while tonic and chronic pain induced lower-frequency GBOs (~55 Hz) predominantly over the prefrontal cortex. Different types of pain and functions of GBOs: Phasic pain-induced GBOs were mainly related to pain perception, tonic pain-related GBOs to multidimensional pain processing, and chronic pain-associated GBOs largely to the pathophysiology of chronic pain.	GBOs hold promise as biomarkers that could inform clinical decisions regarding pain assessment and treatment, but further research is needed.	Limited statistical power due to the small number of studies; Signal quality of GBOs needs to be improved; Heterogeneity and risk of publication bias.	More rigorous research is needed (larger sample size, control for confounding); Investigate GBOs across different pain types to better understand the neuronal basis of pain-related GBOs; Implementation of standardized, advanced data analysis techniques to improve the definition and characterization of GBO temporal, frequential, and spatial features.	GBO magnitude correlates positively with pain intensity in healthy individuals, and these neurophysiological features appear conserved across humans and rodents, more evidence is needed to conclusively determine GBOs’ nature and clinical utility for pain and nociception.
Ahmad and Barkana, 2025 USA [60]	SR	To evaluate methods, participant characteristics, pain states, and brain electrical activity biomarkers in pain research	Fibromyalgia, Sickle cell anemia, chronic knee OA, Pancreatitis, chronic pediatric musculoskeletal pain	Predominantly females, Age range 20–65 (some pediatric cases), Patients had chronic pain for months	EEG biomarkers: theta, alpha, beta, gamma frequency bands, Lower alpha peak frequency, microstates, event-related potentials	Not reported	24	Help differentiate chronic pain from healthy controls (alpha slowing for fibromyalgia, theta increase in OA). Some studies tested whether EEG can predict treatment response, but results are preliminary.	Objective pain measurement, May predict response to medication and neuromodulation, procedure is cheap and non-invasive, can reflect chronicity and severity of pain	Heterogeneity: variable sample size, age range, pain conditions, and mixed methodologies. Most studies were not longitudinal. Results are not standardized for diagnosis or therapy.	Standardization of protocols, longitudinal studies, development of clinical algorithms	EEG biomarkers show promising potential, but findings are not validated for clinical application
Grodzka, 2025, Poland [61]	SR	To review and identify biomarkers which can differentiate primary headache disorders	Migraine with and without aura, cluster headache, tension-type headache, post-traumatic and medication overuse headache	Middle-aged with chronic headache histories, sample sizes are small-to-moderate	CGRP, PACAP, VIP, and inflammatory cytokines (IL-6, TNF-α, hsCRP)	Not reported	21	CGRP, PACAP, and VIP, showed potential in distinguishing migraine, tension-type headache, and cluster headache.	Promising for differentiating headache types and for monitoring disease activity. Have a noninvasive nature (mainly blood or saliva tests).	Biomarkers listed are heterogeneous, small sample sizes, few studies addressed confounders	Future studies need to include more patients, use the same methods, and test the most promising biomarkers. Combining laboratory tests with brain imaging or EEG may help improve accuracy.	Biomarkers for headaches show promise, specifically for telling different headache disorders apart. However, current research is too early, small, and inconsistent to use them in practice.
García-Valdivieso, 2025, Spain [62]	SR &MA	To evaluate whether non-pharmacological analgesia interventions—breastfeeding, skin-to-skin contact, and oral sucrose—reduce pain in newborns	Neonatal procedural pain and stress	Neonates, 28–39 weeks	Cortisol levels	521	10	Cortisol showed changes with pain and analgesia but was too variable to guide diagnosis or treatment directly.	Objective, physiological measure of neonatal stress that complements behavioral pain scores.	Inconsistent study methods.	Larger, standardized studies combining cortisol with other markers can improve neonatal pain assessment.	Cortisol is useful in research but not reliable enough for routine practice; behavioral scales remain the main tool.
Buzhanskyy, 2025, Portugal [63]	SR	Identify neuroimaging biomarkers; map lesion-pain correlations; prognostic roles	Central post-stroke pain	Adults with central post-stroke pain and neuroimaging data	Lesion localizations, functional connectivity patterns	Not reported	14	Potential diagnostic/prognostic value, but not directly linked to treatment	Provides insights into lesion locations; can be potential biomarkers	Small sample size and heterogeneity	Advanced imaging; machine learning integration	Neuroimaging confirms central post-stroke pain mechanisms; more work needed for clinical biomarkers

Abbreviations: A2M—α2-macroglobulin; ACTH—adrenocorticotropic hormone; APF—antiproliferative factor; ASIC—acid-sensing ion channel; BDNF—brain-derived neurotrophic factor; BPS—bladder pain syndrome; CGRP—calcitonin gene-related peptide; CINP—chemotherapy-induced neuropathic pain; CK—creatinine kinase; COMP—cartilage oligomeric matrix protein; COMT—catechol-O-methyltransferase; CPM—conditioned pain modulation; CRH—corticotropin-releasing hormone; CRP—C-reactive protein; CRPS—chronic regional pain syndrome; CT—computed tomography; CTX-I—C-terminal telopeptide of type I collagen; CTX-II—C-terminal cross-linked telopeptide of type II collagen; DOMS—delayed onset muscle soreness; DPN—diabetic peripheral neuropathy; DWI—diffusion weighted imaging; EEG—electroencephalography; EGF—epidermal growth factor; ET-1—endothelin-1; FDG-PET—fluorodeoxyglucose positron emission tomography; fMRI—functional magnetic resonance imaging; GBO—gamma-band oscillations; GCH1—GTP cyclohydrolase 1; GDF-15—growth differentiation factor-15; GF—growth factor; GH—growth hormone; HB-EGF—heparin-binding EGF-like growth factor; HIZ—high-intensity zones; HPA—hypothalamic–pituitary–adrenal; hsCRP—high sensitivity C-reactive protein; IBS—irritable bowel syndrome; IBD—inflammatory bowel disease; IFN—interferon; IGF—insulin-like growth factor; IGF-2—insulin-like growth factor 2; IGL3-25—immunoglobulin lambda chain variable region; IL1RAP—interleukin-1 receptor accessory protein; LBP—low back pain; MA—meta-analysis; MEG—magnetoencephalography; MIF—macrophage migration inhibitory factor; MoA—mechanism of action; MMP—matrix metalloproteinase; MRI—magnetic resonance imaging; MPS—myofascial pain syndrome; MS—multiple sclerosis; MSK—musculoskeletal; N/A—not applicable/not available; NGF—nerve growth factor; NK1R—neurokinin-1 receptor; NIRS—near-infrared spectroscopy; NO—nitric oxide; NOx—nitric oxide metabolites; NSE—neuron-specific enolase; OA—osteoarthritis; OPRM1—mu opioid receptor gene; PACAP—Pituitary Adenylate Cyclase-Activating Polypeptide; PET—positron emission tomography; PFN1—profilin-1; PLP—phantom limb pain; RA—rheumatoid arthritis; RCT—randomized controlled trial; RoB—risk of bias; RoM—range of motion; RSTN—resistin; SAA4—serum amyloid A4; SAM—sympathoadrenal medullary; SAP—serum amyloid P-component; SCD—sickle cell disease; SCI—spinal cord injury; SR—systematic review; T2DM—type 2 diabetes mellitus; TIMP—tissue inhibitor of metalloproteinases; TKR—total knee replacement; TN—trigeminal neuralgia; TNF—tumor necrosis factor; TRPV1—transient receptor potential vanilloid 1; VIP—Vasoactive Intestinal Peptide.

**Table 2:** AMSTAR-2 analysis of included studies.

Citation	1. Did the Research Questions and Inclusion Criteria for the Review Include the Components of PICO?	2. Did the Report of the Review Contain an Explicit Statement that the Review Methods Were Established Prior to the Conduct of the Review and Did the Report Justify Any Significant Deviations from the Protocol?	3. Did the Review Authors Explain Their Selection of the Study Designs for Inclusion in the Review?	4. Did the Review Authors Use a Comprehensive Literature Search Strategy?	5. Did the Review Authors Perform Study Selection in Duplicate?	6. Did the Review Authors Perform Data Extraction in Duplicate?	7. Did the Review Authors Provide a List of Excluded Studies and Justify the Exclusions?	8. Did the Review Authors Describe the Included Studies in Adequate Detail?	9. Did the Review Authors Use a Satisfactory Technique for Assessing the Risk of Bias (RoB) in Individual Studies that Were Included in the Review?	10. Did the Review Authors Report on the Sources of Funding for the Studies Included in the Review?	11. If Meta-Analysis Was Performed Did the Review Authors Use Appropriate Methods for Statistical Combination of Results?	12. If meta-Analysis Was Performed, Did the Review Authors Assess the Potential Impact of RoB in Individual Studies on the Results of the Meta-Analysis or Other Evidence Synthesis?	13. Did the Review Authors Account for RoB in Individual Studies When Interpreting/Discussing the Results of the Review?	14. Did the Review Authors Provide a Satisfactory Explanation for, and Discussion of, Any Heterogeneity Observed in the Results of the Review?	15. If They Performed Quantitative Synthesis Did the Review Authors Carry Out Adequate Investigation of Publication Bias (Small Study Bias) and Discuss Its Likely Impact on the Results of the Review?	16. Did the Review Authors Report Any Potential Sources of Conflict of Interest, Including Any Funding They Received for Conducting the Review?
Pinto 2023 [56]	+	+	−	Partial yes	+	+	−	+	+	−	N/A	N/A	N/A	N/A	N/A	+
Saravanan 2023 [57]	+	−	−	Partial yes	+	−	−	Partial yes	+	−	N/A	N/A	N/A	N/A	N/A	+
Beiner 2023 [58]	+	+	−	Partial yes	+	+	+	+	Partial yes	−	+	−	+	+	+	+
Zebhauser 2023 [15]	+	+	−	Partial yes	+	−	−	Partial yes	+	−	N/A	N/A	N/A	N/A	N/A	+
Sanabria-Mazo 2022 [16]	+	+	+	+	+	+	+	+	+	−	N/A	N/A	N/A	N/A	N/A	+
Gomez-Pilar 2022 [17]	+	−	−	Partial yes	+	NG	−	Partial yes	−	−	N/A	N/A	N/A	N/A	N/A	+
Matesanz-García 2022 [18]	+	+	+	Partial yes	+	+	−	Could not access supp. data	+	−	N/A	N/A	N/A	N/A	N/A	+
Mussigmann 2022 [19]	+	−	−	Partial yes	+	+	−	Partial yes	+	−	N/A	N/A	N/A	N/A	N/A	+
Andronic 2022 [20]	+	+	+	Partial yes	+	+	+	Partial yes	+	−	N/A	N/A	N/A	N/A	N/A	+
Kumbhare 2021 [21]	+	−	+	Partial yes	+	+	+	Partial yes	Partial yes	−	+	−	−	+	+	+
Baka 2021 [22]	+	−	−	Partial yes	+	+	−	Partial yes	Partial yes	−	+	−	+	+	−	+
Bonifácio de Assis 2021 [23]	N/A—scoping review															
Aroke & Powell-Roach, 2020 [24]	+	−	−	Partial yes	+	NG	−	Partial yes	−	−	N/A	N/A	N/A	N/A	N/A	+
Morris 2020 [25]	+	−	−	Partial yes	+	+	+	+	+	−	N/A	N/A	N/A	N/A	N/A	+
Vadasz 2020 [26]	+	−	−	Partial yes	NG	+	−	− (nothing about population)	−	−	N/A	N/A	N/A	N/A	N/A	−
Lim 2020 [27]	+	−	−	Partial yes	+	+	+	+	+	−	N/A	N/A	N/A	N/A	N/A	+
Henssen 2019 [28]	+	−	−	Partial yes	+	+	−	Partial yes	−	−	− (no CIs)	−	−	−	−	+
Magalhaes 2019 [29]	+	−	−	Partial yes	+	NG	−	Partial yes	−	−	N/A	N/A	N/A	N/A	N/A	−
Fernandes 2019 [30]	+	+	+	Partial yes	+	+	−	Partial yes	+	−	N/A	N/A	N/A	N/A	N/A	+
Andronic 2020 [31]	+	+	−	Partial yes	+	NG	−	Partial yes	+	−	N/A	N/A	N/A	N/A	N/A	+
Ping 2019 [32]	+	−	+	Partial yes	+	+	−	Partial yes	−	−	N/A	N/A	N/A	N/A	N/A	+
Jungen 2019 [33]	+	−	+	Partial yes	+	+	+	Partial yes	+	−	N/A	N/A	N/A	N/A	N/A	+
Gardner 2019 [34]	+	−	−	Partial yes	+	NG	−	Partial yes	−	−	N/A	N/A	N/A	N/A	N/A	+
Teraguchi 2018 [35]	+	−	−	Partial yes	+	+	−	Partial yes	−	−	N/A	N/A	N/A	N/A	N/A	+
van den Berg 2018 [36]	+	+	−	Partial yes	+	+	−	Partial yes	+	−	N/A	N/A	N/A	N/A	N/A	+
Andrade 2018 [37]	+	+	−	Partial yes	+	+	−	Partial yes	+	−	N/A	N/A	N/A	N/A	N/A	+
Wallwork 2017 [38]	+	−	−	Partial yes	+	+	+	Partial yes	Partial yes	−	+	−	−	+	−	+
Bjorland 2016 [39]	+	+	−	Partial yes	+	+	+	+	+	−	N/A	N/A	N/A	N/A	N/A	+
Gold 2016 [40]	+	−	−	Partial yes	+	NG	−	Partial yes	+	−	N/A	N/A	N/A	N/A	N/A	+
Dell’Isola 2016 [41]	+	−	−	− (one database)	+	+	−	Partial yes	+	−	N/A	N/A	N/A	N/A	N/A	+
Akinci 2016 [42]	N/A—narrative review															
Kawi 2016 [43]	+	−	−	Partial yes	NG	NG	−	+	+	−	N/A	N/A	N/A	N/A	N/A	+
Nepple 2015 [44]	+	−	−	Partial yes	+	+	+	Partial yes	−	−	N/A	N/A	N/A	N/A	N/A	+
Jin 2015 [45]	+	−	−	+	+	+	+	Partial yes	Partial yes	−	+	−	−	+	+	+
Hunter 2011 [46]	+	−	−	Partial yes	NG	+	−	Partial yes	Partial yes	−	N/A	N/A	N/A	N/A	N/A	+
Mauri, 2025 [47]	+	+	−	Partial Yes	+	+	+	Partial Yes	−	−	+	+	+	+	+	+
Lozano-Parra, 2024 [48]	+	+	−	Partial Yes	+	−	+	Partial Yes	+	−	No MA	No MA	−	−	No MA	+
Rodrigues, 2024 [49]	+	+	−	Partial Yes	+	+	+	+	Partial Yes	+	No MA	No MA	+	+	No MA	−
Gkouvi, 2024 [50]	−	+	−	Partial Yes	+	+	+	Partial Yes	−	+	No MA	No MA	−	−	No MA	+
Sima, 2024 [51]	+	+	−	Partial Yes	+	+	−	Partial Yes	+	−	+	+	−	+	−	+
Puerto Valencia, 2024 [52]	+	−	−	Partial Yes	+	−	+	Partial Yes	+	−	No MA	No MA	−	+	No MA	+
He, 2024 [53]	+	+	−	Partial Yes	+	+	Partial Yes	+	−	−	+	+	−	+	−	+
Yang, 2024 [54]	−	−	−	Partial Yes	+	+	Partial Yes	Partial Yes	−	−	+	+	−	+	−	−
Søborg, 2024 [55]	+	+	−	Partial Yes	+	−	Partial Yes	Partial Yes	+	−	No MA	No MA	+	+	No MA	+
Li, 2023 [59]	−	+	−	Partial Yes	+	−	Partial Yes	Partial Yes	+	−	+	+	−	+	+	+
Ahmad, 2025 [60]	+	+	Partial Yes	+	Partial Yes	Partial Yes	Partial Yes	+	+	+	No MA	No MA	+	+	No MA	+
Grodzka, 2025 [61]	+	+	+	+	+	+	Partial Yes	+	+	+	No MA	No MA	+	+	No MA	+
García-Valdivieso [62]	+	+	+	+	+	Partial Yes	Partial Yes	+	+	+	+	Partial Yes	+	+	+	+
Buzhanskyy, 2025 [63]	Partial Yes	+	Partial Yes	Partial Yes	Partial Yes	Partial Yes	Partial Yes	+	+	+	No MA	No MA	+	+	No MA	+

Abbreviations: MA—meta-analysis; N/A—not applicable.

## Data Availability

No new data were created or analyzed in this study.

## Supplementary Materials

The following supporting information can be downloaded at: https://www.mdpi.com/article/10.3390/jcm15020550/s1, Table S1: PRISMA checklist.

## Abbreviations

The following abbreviations are used in this manuscript:

PRISMA	Preferred Reporting Items for Systematic Reviews and Meta-Analyses
SR	Systematic Review
MA	Meta-Analysis
RCT	Randomized Controlled Trial
AMSTAR-2	A Measurement Tool to Assess Systematic Reviews, version 2
MRI	Magnetic Resonance Imaging
PET	Positron Emission Tomography
LBP	Low Back Pain
NsLBP	Non-specific Low Back Pain
CLBP	Chronic Low Back Pain
OA	Osteoarthritis
AS	Ankylosing Spondylitis
RA	Rheumatoid Arthritis
MS	Multiple Sclerosis
BMS	Burning Mouth Syndrome
BPS	Bladder Pain Syndrome
CRPS	Complex Regional Pain Syndrome
MPS	Myofascial Pain Syndrome
DOMS	Delayed Onset Muscle Soreness
DPN	Diabetic Peripheral Neuropathy
HIZ	High-Intensity Zone
CRP	C-reactive Protein
IL-6	Interleukin-6
IL-8	Interleukin-8
IL-4	Interleukin-4
TNF-α	Tumor Necrosis Factor-alpha
BDNF	Brain-Derived Neurotrophic Factor
MIF	Macrophage Migration Inhibitory Factor
CGRP	Calcitonin Gene-Related Peptide
HPA axis	Hypothalamic–Pituitary–Adrenal axis
CTX-II	C-terminal Cross-linked Telopeptide of Type II Collagen
COMP	Cartilage Oligomeric Matrix Protein
CPM	Conditioned Pain Modulation
EEG	Electroencephalography
MEG	Magnetoencephalography
GBO/GBOs	Gamma-Band Oscillation(s)
OPRM1	Opioid Receptor Mu 1 gene
COMT	Catechol-O-Methyltransferase gene

## References

[1] Goldberg D.S., McGee S.J. (2011). Pain as a global public health priority. BMC Public Health.

[2] Dorner T.E. (2018). Pain and chronic pain epidemiology: Implications for clinical and public health fields. Wien. Klin. Wochenschr..

[3] Mills S.E.E., Nicolson K.P., Smith B.H. (2019). Chronic pain: A review of its epidemiology and associated factors in population-based studies. Br. J. Anaesth..

[4] Docking R.E., Fleming J., Brayne C., Zhao J., Macfarlane G.J., Jones G.T., Cambridge City over-75s Cohort Study collaboration (2011). Epidemiology of back pain in older adults: Prevalence and risk factors for back pain onset. Rheumatology.

[5] Thomas E., Peat G., Harris L., Wilkie R., Croft P.R. (2004). The prevalence of pain and pain interference in a general population of older adults: Cross-sectional findings from the North Staffordshire Osteoarthritis Project (NorStOP). Pain.

[6] Janevic M.R., McLaughlin S.J., Heapy A.A., Thacker C., Piette J.D. (2017). Racial and Socioeconomic Disparities in Disabling Chronic Pain: Findings From the Health and Retirement Study. J. Pain.

[7] Maniadakis N., Gray A. (2000). The economic burden of back pain in the UK. Pain.

[8] Swedish Council on Health Technology Assessment (2006). *Methods of Treating Chronic Pain: A Systematic Review*.

[9] Ricci J.A., Stewart W.F., Chee E., Leotta C., Foley K., Hochberg M.C. (2005). Pain exacerbation as a major source of lost productive time in US workers with arthritis. Arthritis Rheum..

[10] Eldabe S., Obara I., Panwar C., Caraway D. (2022). Biomarkers for Chronic Pain: Significance and Summary of Recent Advances. Pain Res. Manag..

[11] Ou F.S., Michiels S., Shyr Y., Adjei A.A., Oberg A.L. (2021). Biomarker Discovery and Validation: Statistical Considerations. J. Thorac. Oncol..

[12] Larsson A., Bäckryd E., Eriksson M. (2023). Biomarkers in Pain. Biomedicines.

[13] Page M.J., McKenzie J.E., Bossuyt P.M., Boutron I., Hoffmann T.C., Mulrow C.D., Shamseer L., Tetzlaff J.M., Akl E.A., Brennan S.E. (2021). The PRISMA 2020 statement: An updated guideline for reporting systematic reviews. BMJ.

[14] Shea B.J., Reeves B.C., Wells G., Thuku M., Hamel C., Moran J., Moher D., Tugwell P., Welch V., Kristjansson E. (2017). AMSTAR 2: A critical appraisal tool for systematic reviews that include randomised or non-randomised studies of healthcare interventions, or both. BMJ.

[15] Zebhauser P.T., Hohn V.D., Ploner M. (2023). Resting-state electroencephalography and magnetoencephalography as biomarkers of chronic pain: A systematic review. Pain.

[16] Sanabria-Mazo J.P., Colomer-Carbonell A., Carmona-Cervelló M., Feliu-Soler A., Borràs X., Grasa M., Esteve M., Maes M., Edo S., Sanz A. (2022). Immune-inflammatory and hypothalamic-pituitary-adrenal axis biomarkers are altered in patients with non-specific low back pain: A systematic review. Front. Immunol..

[17] Gomez-Pilar J., Martínez-Cagigal V., García-Azorín D., Gómez C., Guerrero Á., Hornero R. (2022). Headache-related circuits and high frequencies evaluated by EEG, MRI, PET as potential biomarkers to differentiate chronic and episodic migraine: Evidence from a systematic review. J. Headache Pain.

[18] Matesanz-García L., Schmid A.B., Cáceres-Pajuelo J.E., Cuenca-Martínez F., Arribas-Romano A., González-Zamorano Y., Goicoechea-García C., Fernández-Carnero J. (2022). Effect of Physiotherapeutic Interventions on Biomarkers of Neuropathic Pain: A Systematic Review of Preclinical Literature. J. Pain.

[19] Mussigmann T., Bardel B., Lefaucheur J.P. (2022). Resting-state electroencephalography (EEG) biomarkers of chronic neuropathic pain. A systematic review. NeuroImage.

[20] Andronic D., Andronic O., Juengel A., Berli M.C., Distler O., Brunner F. (2022). Skin biomarkers associated with complex regional pain syndrome (CRPS) Type I: A systematic review. Rheumatol. Int..

[21] Kumbhare D., Hassan S., Diep D., Duarte F.C.K., Hung J., Damodara S., West D.W.D., Selvaganapathy P.R. (2022). Potential role of blood biomarkers in patients with fibromyalgia: A systematic review with meta-analysis. Pain.

[22] Baka P., Escolano-Lozano F., Birklein F. (2021). Systemic inflammatory biomarkers in painful diabetic neuropathy. J. Diabetes Its Complicat..

[23] Bonifácio De Assis E., Dias De Carvalho C., Martins C., Andrade S. (2021). Beta-Endorphin as a Biomarker in the Treatment of Chronic Pain with Non-Invasive Brain Stimulation: A Systematic Scoping Review. J. Pain Res..

[24] Aroke E.N., Powell-Roach K.L. (2020). The Metabolomics of Chronic Pain Conditions: A Systematic Review. Biol. Res. Nurs..

[25] Morris P., Ali K., Merritt M., Pelletier J., Macedo L.G. (2020). A systematic review of the role of inflammatory biomarkers in acute, subacute and chronic non-specific low back pain. BMC Musculoskelet. Disord..

[26] Vadasz B., Gohari J., West D.W., Grosman-Rimon L., Wright E., Ozcakar L., Srbely J., Kumbhare D. (2020). Improving characterization and diagnosis quality of myofascial pain syndrome: A systematic review of the clinical and biomarker overlap with delayed onset muscle soreness. Eur. J. Phys. Rehabil. Med..

[27] Lim Y.Z., Wang Y., Cicuttini F.M., Hughes H.J., Chou L., Urquhart D.M., Ong P.X., Hussain S.M. (2020). Association Between Inflammatory Biomarkers and Nonspecific Low Back Pain: A Systematic Review. Clin. J. Pain.

[28] Henssen D., Dijk J., Knepflé R., Sieffers M., Winter A., Vissers K. (2019). Alterations in grey matter density and functional connectivity in trigeminal neuropathic pain and trigeminal neuralgia: A systematic review and meta-analysis. NeuroImage Clin..

[29] Magalhaes T.F., Baracat E.C., Doumouchtsis S.K., Haddad J.M. (2019). Biomarkers in the diagnosis and symptom assessment of patients with bladder pain syndrome: A systematic review. Int. Urogynecol. J..

[30] Fernandes C., Pidal-Miranda M., Samartin-Veiga N., Carrillo-de-la-Peña M.T. (2019). Conditioned pain modulation as a biomarker of chronic pain: A systematic review of its concurrent validity. Pain.

[31] Andronic O., Ernstbrunner L., Jüngel A., Wieser K., Bouaicha S. (2020). Biomarkers associated with idiopathic frozen shoulder: A systematic review. Connect. Tissue Res..

[32] Ping Z., Wen Z., Jinhua L., Jinghe L. (2019). Research on central sensitization of endometriosis-associated pain: A systematic review of the literature. J. Pain Res..

[33] Jungen M.J., Ter Meulen B.C., Van Osch T., Weinstein H.C., Ostelo R.W.J.G. (2019). Inflammatory biomarkers in patients with sciatica: A systematic review. BMC Musculoskelet. Disord..

[34] Gardner K., Laird B.J.A., Fallon M.T., Sande T.A. (2019). A systematic review examining clinical markers and biomarkers of analgesic response to radiotherapy for cancer-induced bone pain. Crit. Rev. Oncol. Hematol..

[35] Teraguchi M., Yim R., Cheung J.P.Y., Samartzis D. (2018). The association of high-intensity zones on MRI and low back pain: A systematic review. Scoliosis.

[36] Van Den Berg R., Jongbloed E.M., De Schepper E.I.T., Bierma-Zeinstra S.M.A., Koes B.W., Luijsterburg P.A.J. (2018). The association between pro-inflammatory biomarkers and nonspecific low back pain: A systematic review. Spine J..

[37] Andrade A., Vilarino G.T., Sieczkowska S.M., Coimbra D.R., Steffens R.D.A.K., Vietta G.G. (2018). Acute effects of physical exercises on the inflammatory markers of patients with fibromyalgia syndrome: A systematic review. J. Neuroimmunol..

[38] Wallwork S.B., Grabherr L., O’Connell N.E., Catley M.J., Moseley G.L. (2017). Defensive reflexes in people with pain–a biomarker of the need to protect? A meta-analytical systematic review. Rev. Neurosci..

[39] Bjorland S., Moen A., Schistad E., Gjerstad J., Røe C. (2016). Genes associated with persistent lumbar radicular pain; a systematic review. BMC Musculoskelet. Disord..

[40] Gold J.E., Hallman D.M., Hellström F., Björklund M., Crenshaw A.G., Mathiassen S.E., Barbe M.F., Ali S. (2017). Systematic review of quantitative imaging biomarkers for neck and shoulder musculoskeletal disorders. BMC Musculoskelet. Disord..

[41] Dell’Isola A., Allan R., Smith S.L., Marreiros S.S.P., Steultjens M. (2016). Identification of clinical phenotypes in knee osteoarthritis: A systematic review of the literature. BMC Musculoskelet. Disord..

[42] Akinci A., Al Shaker M., Chang M.H., Cheung C.W., Danilov A., José Dueñas H., Kim Y.C., Guillen R., Tassanawipas W., Treuer T. (2016). Predictive factors and clinical biomarkers for treatment in patients with chronic pain caused by osteoarthritis with a central sensitisation component. Int. J. Clin. Pract..

[43] Kawi J., Lukkahatai N., Inouye J., Thomason D., Connelly K. (2016). Effects of Exercise on Select Biomarkers and Associated Outcomes in Chronic Pain Conditions: Systematic Review. Biol. Res. Nurs..

[44] Nepple J.J., Thomason K.M., An T.W., Harris-Hayes M., Clohisy J.C. (2015). What Is the Utility of Biomarkers for Assessing the Pathophysiology of Hip Osteoarthritis? A Systematic Review. Clin. Orthop. Relat. Res..

[45] Jin X., Beguerie J.R., Zhang W., Blizzard L., Otahal P., Jones G., Ding C. (2015). Circulating C reactive protein in osteoarthritis: A systematic review and meta-analysis. Ann. Rheum. Dis..

[46] Hunter D.J., Zhang W., Conaghan P.G., Hirko K., Menashe L., Li L., Reichmann W.M., Losina E. (2011). Systematic review of the concurrent and predictive validity of MRI biomarkers in OA. Osteoarthr. Cartil..

[47] Mauri C., Cerulli C., Grazioli E., Minganti C., Tranchita E., Scotto Di Palumbo A., Parisi A. (2025). Role of exercise on pain, functional capacity, and inflammatory biomarkers in osteoarthritis: A systematic review and meta-analysis. Ann. Phys. Rehabil. Med..

[48] Lozano-Parra A., Herrera V., Urcuqui-Inchima S., Ramírez R.M.G., Villar L.Á. (2024). Acute Immunological Profile and Prognostic Biomarkers of PersistentJoint Pain in Chikungunya Fever: A Systematic Review. Yale J. Biol. Med..

[49] Rodrigues R., Mesquita C.M., Alves H.B.D.N., Silva F.G., Vieira W.D.A., Aguiar P.C.S.D., Flores-Mir C., Paranhos L.R., Brito-Júnior R.B. (2024). Changes in salivary biomarkers of pain, anxiety, stress, and inflammation related to tooth movement during orthodontic treatment: A systematic review. Dent. Press J. Orthod..

[50] Gkouvi A., Tsiogkas S.G., Bogdanos D.P., Gika H., Goulis D.G., Grammatikopoulou M.G. (2024). Proteomics in Patients with Fibromyalgia Syndrome: A Systematic Review of Observational Studies. Curr. Pain Headache Rep..

[51] Sima S., Chen X., Diwan A.D. (2024). The association between inflammatory biomarkers and low back disorder: A systematic review and meta-analysis. Biomarkers.

[52] Puerto Valencia L.M., He Y., Wippert P.M. (2024). The changes of blood-based inflammatory biomarkers after non-pharmacologic interventions for chronic low back pain: A systematic review. BMC Musculoskelet. Disord..

[53] He M., Huoshen W., Li X., Sun C. (2024). Salivary and serum biomarkers to evaluate psychological disorders in burning mouth syndrome: A systematic review and meta-analysis. J. Oral Pathol. Med..

[54] Yang C.X., Yu Z.R., Li G., Liang X.H., Li C.D. (2024). Gray Matter Abnormalities in Patients with Chronic Low Back Pain: A Systematic Review and Meta-Analysis of Voxel-Based Morphometry Studies. World Neurosurg..

[55] Søborg M.K., Jensen R.H., Barloese M., Petersen A.S. (2024). Biomarkers in cluster headache: A systematic review. Headache.

[56] Pinto E.M., Neves J.R., Laranjeira M., Reis J. (2023). The importance of inflammatory biomarkers in non-specific acute and chronic low back pain: A systematic review. Eur. Spine J..

[57] Saravanan A., Bai J., Bajaj P., Sterner E., Rajagopal M., Sanders S., Luckose A., Kushnick M., Starkweather A. (2023). Composite Biomarkers, Behavioral Symptoms, and Comorbidities in Axial Low Back Pain: A Systematic Review. Biol. Res. Nurs..

[58] Beiner E., Lucas V., Reichert J., Buhai D.V., Jesinghaus M., Vock S., Drusko A., Baumeister D., Eich W., Friederich H.C. (2023). Stress biomarkers in individuals with fibromyalgia syndrome: A systematic review with meta-analysis. Pain.

[59] Li Z., Zhang L., Zeng Y., Zhao Q., Hu L. (2023). Gamma-band oscillations of pain and nociception: A systematic review and meta-analysis of human and rodent studies. Neurosci. Biobehav. Rev..

[60] Ahmad B., Barkana B.D. (2025). Pain and the Brain: A Systematic Review of Methods, EEG Biomarkers, Limitations, and Future Directions. Neurol. Int..

[61] Grodzka O., Łagowski W., Eyileten C., Domitrz I. (2025). Biomarkers in headaches as a potential solution to simplify differential diagnosis of primary headache disorders: A systematic review. J. Headache Pain.

[62] García-Valdivieso I., Sánchez-Infante J., Pando Cerra P., Yáñez-Araque B., Hernández-Iglesias S., Peña Cambón F., Álvarez-Bueno C., Checa Peñalver A., Pérez-Pozuelo J.M., Gómez-Cantarino S. (2025). Assessment of cortisol as a neonatal pain biomarker in the application of non-pharmacological analgesia therapies: Systematic review and meta-analysis. BMC Pediatr..

[63] Buzhanskyy A., Duarte I.C., Patto A.V., Donato H., Castelo-Branco M., Abejas A., Lapa T. (2025). Neuroimage Signature in Post-Stroke Pain: A Systematic Review. Curr. Pain Headache Rep..

